# Health benefits of ethnic fermented foods

**DOI:** 10.3389/fnut.2025.1677478

**Published:** 2025-09-26

**Authors:** Dushica Santa, Melanie Huch, Dominic A. Stoll, Hülya Cunedioglu, Reimo Priidik, Barçın Karakaş-Budak, Antonia Matalas, Vincenzo Pennone, Aiswarya Girija, Elena Arranz, Michail Syrpas, Arghya Mukherjee, Paul D. Cotter, Sandra Mojsova, Christophe Chassard, Smilja Praćer, Guy Vergères, Mary-Liis Kütt

**Affiliations:** ^1^Faculty of Agricultural Sciences and Food, Ss. Cyril and Methodius University in Skopje, Skopje, North Macedonia; ^2^Department of Safety and Quality of Fruit and Vegetables, Max Rubner-Institut, Karlsruhe, Germany; ^3^Department of Agriculture, Food and Natural Science Engineering, University of Foggia, Foggia, Italy; ^4^Scienzanova srl, Termoli, Italy; ^5^Wizon OÜ, Tallinn, Estonia; ^6^Department of Food Engineering, Akdeniz University, Faculty of Engineering, Antalya, Türkiye; ^7^School of Health Sciences and Education, Harokopio University, Athens, Greece; ^8^Cell and Tissue Engineering Laboratory, IRCCS Istituto Ortopedico Galeazzi, Milan, Italy; ^9^Institute of Biological, Environmental & Rural Sciences (IBERS), Aberystwyth University, Plas Gogerddan, Aberystwyth, Wales, United Kingdom; ^10^Universidad Autónoma de Madrid (CEI UAM+CSIC), Institute of Food Science Research (CIAL, CSIC-UAM), Madrid, Spain; ^11^Department of Food Science and Technology, Kaunas University of Technology, Kaunas, Lithuania; ^12^Department of Food Biosciences, Teagasc, Cork, Ireland; ^13^APC Microbiome Ireland, Cork, Ireland; ^14^VistaMilk, Cork, Ireland; ^15^Faculty of Veterinary Medicine, Ss. Cyril and Methodius University in Skopje, Skopje, North Macedonia; ^16^UCA, INRAE, VetAgro Sup, UMRF 0545, Clermont-Ferrand, France; ^17^Institute for Biological Research Siniša Stanković, National Institute of the Republic of Serbia, University of Belgrade, Belgrade, Serbia; ^18^Agroscope, Bern, Switzerland; ^19^äio Tech OÜ, Tallinn, Estonia

**Keywords:** ethnic fermented foods, fermentation, diversity, lactic acid bacteria, bioactive compounds

## Abstract

Food science and technology have evolved to enhance food availability, nutrition, safety, and marketability, addressing global challenges such as hunger and malnutrition. However, projections indicate that by 2030, over 670 million people may face hunger, emphasizing the need for innovative and sustainable solutions. Ethnic fermented foods (EFF) originate from the heritage and culture of various ethnic groups who utilize their ethnomicrobiological knowledge of fermentation with local plant- or animal-based ingredients. EFF provide a promising approach to promote a healthier, sustainable, and more inclusive global food system. They are also traditionally produced by combining local ingredients with artisanal practices. However, urbanization and changes in eating habits threaten the preservation of EFF and the associated native microorganisms that are essential for their production. This research systematically examines health-related endpoints reported in human clinical studies of EFF. While some EFF such as natto, kimchi, and tempeh, originating from Asia have gained global recognition and consumption, multiple others remain unknown and understudied. This comprehensive narrative review aims to identify the potential health benefits associated with these foods. A systematic search in PubMed, Scopus, and the Cochrane Library Central initially resulted in 3,488 entries, which were narrowed down to 125 reports fulfilling the selection criteria defined to identify human studies investigating the functionality of EFF. CADIMA software was used to manage and select references. Data synthesis focused on clinical indications, offering valuable insights into health benefits of EFF, such as gut health, immune modulation, metabolic benefit, cognitive function, cardiovascular, bone, oral, eye, skin health and antimicrobial effect. This systematic narrative review highlights the importance of EFF in improving health, emphasizing their role in sustainable food systems, and underscoring the importance of preserving traditional practices.

**Systematic review registration:**

https://osf.io/hnksr/.

## 1 Introduction

Fermentation has been used for centuries to preserve food and extend the shelf life of raw ingredients. Initially, fermentation likely occurred by accident, when plant- and animal-based raw materials fermented spontaneously. Over time, human civilizations across the world began to intentionally harness the processes utilizing local food sources, indigenous microbiota, and unique environmental conditions to create a diverse array of fermented foods. Societies recognized the benefits of spontaneous fermentation, which today is mainly carried out under controlled conditions and environments. The diversity of fermented foods and the technologies used to produce them are as varied as the cultures in which they are embedded (1). According to Lee et al. (2), referencing earlier classifications, fermented foods can be grouped by the dominant fermentation pathways, such as alcohol, acid, carbon dioxide, and amino acid/peptide fermentations.

During fermentation, sugars are primarily degraded by microorganisms such as bacteria, yeasts, and molds into various food components and secondary metabolites. These fermentation-derived compounds not only ensure food safety and extend shelf life but also enhance the bioavailability and absorption of several nutrients. Furthermore, fermentation can improve the flavor, texture, and nutritional value of foods compared to their unfermented counterparts. Fermented foods serve as important sources of vital nutrients and have also shaped the human microbiome. In this respect, fermented foods can be considered drivers of human evolution (3). Studies from different continents have shown associations between microorganisms present in certain fermented foods (e.g., kefir, yeast, kombucha, chungkookjang, cheeses, and fermented vegetables) and beneficial health outcomes, such as weight maintenance, reduced risk of cardiovascular disease, antidiabetic and anti-constipation effects, improvements in glucose and lipid levels, stimulation of the immune system, anticarcinogenic effects, and, importantly, reduced mortality (4). Recent large-scale genome-wide analyses have demonstrated that lactic acid bacteria (LAB) present in fermented foods are also found in the human gut, providing strong evidence that fermented foods are a potential source of LAB for the gut microbiome (5).

Fermentation processes occur across the world regardless of temperature, season, or type of raw material used. Although modern fermentation is typically strictly controlled to ensure safety and sensory stability, ethnic fermented foods (EFF) remain unique products of local traditions. By definition, “ethnic” refers to a population subgroup with a shared national or cultural tradition. In the context of food, ethnic foods originate from the culinary traditions of specific cultural or regional groups, often distinct from mainstream cuisine. Defining ethnic fermented foods (EFF) helps distinguish them from other fermented products. Ethnic fermented foods can therefore be defined as foods that come from the heritage and culture of an ethnic group, where people apply their own traditional microbiological knowledge of fermentation together with local plant or animal ingredients (6, 7). Perricone et al. (8) provide a comprehensive overview of the raw materials used for EFF, such as cereals, legumes, milk, fish, and meat. Tamang and Thapa (7) further elaborate on EFF from both regional and starter culture perspectives, describing fermented foods across Africa, Asia, Australia, Europe, North America, and South America. From the perspective of starter cultures, both traditional and modern processes rely predominantly on LAB, non-lactic acid bacteria such as *Bacillaceae* of Bacillota, yeasts, and filamentous fungi (7).

Ethnic fermented foods have deep cultural roots in many parts of the world. Northeast Asia, particularly the Korean Peninsula, has been identified as one of the early centers of fermentation technology, linked to the invention and use of earthenware during the Primitive Pottery Age (8000-3,000 BCE) (2). Southeast Asia shows exceptional diversity in indigenous fermented foods made from cereals, legumes, vegetables, fish, and meat, reflecting local resources and traditions (9). In Africa, a wide range of fermented foods, such as cereal-based beverages, fermented dairy, and root-based products, play a vital role in nutrition, cultural heritage, and food security (10). These regions not only represent cultural richness but also contribute a substantial share of the available human studies on EFF included in this review.

Many of the most widely recognized EFF originate from Asia. In recent years, EFF products such as kimchi, tempeh, natto, miso, lassi, shoyu, dahi, and amazake have gained popularity beyond their regions of origin, becoming increasingly integrated into daily diets (11). While traditional fermented foods from India and other Asian regions are deeply embedded in local diets and linked to potential probiotic and nutritional benefits, there is still a scarcity of clinical evidence confirming their effectiveness in humans (12, 139). In Africa, cereals such as sorghum, millet, and maize serve as the primary raw materials for fermented foods like ogi, injera, kisra, fufu, and gari (13, 14). Europe is known for its wide variety of fermented dairy products, with each country offering yogurt-like products made from cow, sheep, or goat milk, as well as a tradition of fermented meat products such as salami. Traditional fermented foods commonly found in European diets include tarhana, buttermilk, skyr, kefir, koumiss, viili, chorizo, salami, pepperoni, and table olives (15, 16). Although EFF from North America are less frequently reported, human intervention studies have been conducted on products such as buttermilk (17), poi (18), and tempeh (19), highlighting the continent’s cultural and dietary diversity.

In the 21st century, globalization and digital communication have transformed food systems and consumer perceptions (20). Increased access to information and the exchange of cultural practices have influenced daily life and dietary habits. Traditional fermented foods are now gaining interest beyond their regions of origin, partly due to the perceived novelty and associated health benefits. However, despite their growing popularity, many EFF remain underrepresented in health research. Most scientific evidence focuses on a few well-known products like kimchi, natto, and tempeh, while many other traditional fermented foods lack systematic health assessments.

Previous reviews on EFF have largely taken a narrative approach, focusing on product diversity, cultural heritage, and microbial ecology. For example, Perricone et al. (8) mapped EFF globally, describing origins, raw materials, production methods, and dominant microorganisms across different substrates. Tamang and Thapa (7) compiled regional varieties and starter culture practices, emphasizing microbial diversity. Lee et al. (2) classified EFF by fermentation type, raw materials, microorganisms, and regional practices. None of these reviews focused on clinical studies assessing the impact of EFF on human health.

This systematic narrative review aims to address this gap by identifying the potential health benefits associated with EFF, in particular through the analysis of the clinical endpoints and associated bioactive compounds reported in human intervention and observational studies. The review also explores whether the health benefits arise from raw material composition, the characteristics of the starter culture, or transformations that occur during fermentation. Where available, mechanisms of action are also discussed.

This review is part of the activities developed by the COST Action CA20128 “Promoting innovation of fermented foods” (PIMENTO) which coordinates 16 complementary systematic reviews targeting either specific health areas for fermented foods or the health properties of specific fermented foods or food groups (21). In line with this strategy, this review focuses exclusively on human clinical studies having investigated EFF to provide a targeted, evidence-based synthesis of the available data not previously reported in the literature.

Through this work, we seek to systematically map the current scientific knowledge about the health benefits of EFF and highlight existing research gaps, to ultimately support the preservation and promotion of traditional food practices within sustainable and healthy food systems.

## 2 Methods

This systematic narrative review was conducted following a predefined protocol developed by the PIMENTO COST Action and is registered on the open science framework: https://osf.io/hnksr/. The protocol follows the guidance of Muka et al. (22) and the PRISMA guidelines (23), adapted to include both systematic and non-systematic components.

### 2.1 Study selection and data extraction

#### 2.1.1 Study protocol

The methodology for this review is based on the framework for systematic reviews proposed by Muka et al. (22), the PRISMA guidelines (23), and the PIMENTO Study Protocol (PIMENTO-SP) (21). The study protocol is deposited in the Open Science Framework (OSF) and publicly available at: https://osf.io/hnksr/.

#### 2.1.2 Literature search

**Figure 1:**
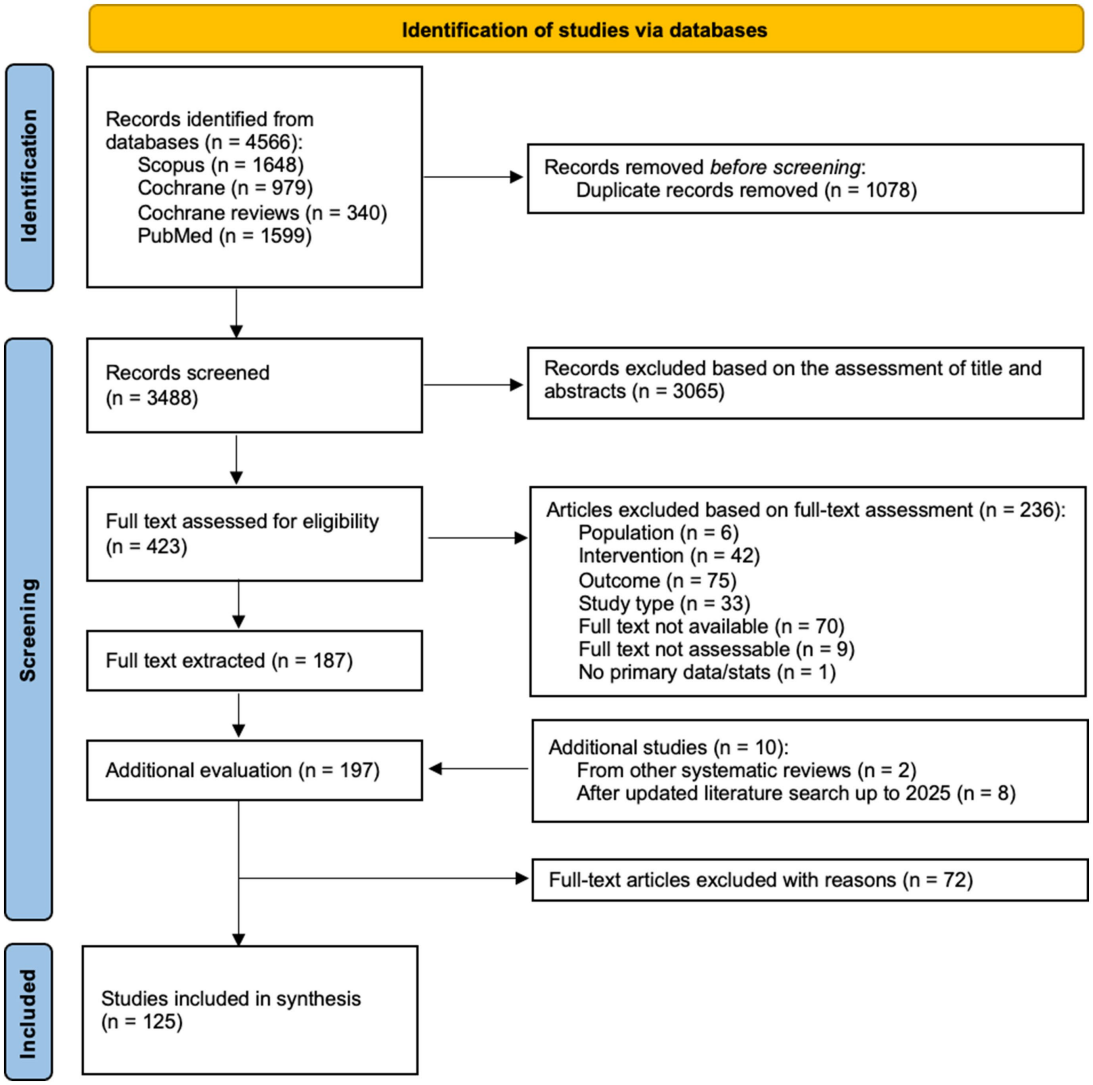
PRISMA flow diagram summarizing the study identification and selection process.

A systematic literature search was conducted to identify human studies assessing the health-associated endpoints reported for EFF in human clinical studies. The search covered three databases: PubMed, Scopus, and the Cochrane library and outputs from January 1, 1970, to December 31, 2024, were considered for this study (Figure 1). The search strategy was based on the PIMENTO-SP. The search strings were tailored to identify EFF from various food groups (dairy, legume, cereal, vegetable, fish, and meat). The list of EFF terms was based on Tamang and Thapa (7) and Perricone et al. (8). Search terms included fermented foods from different regions and cultures (Supplementary Table S1). Products classified as alcoholic beverages with an ethanol content >1.25%, non-fermented pickled foods, or supplements (capsules/pills) were excluded.

#### 2.1.3 Study selection criteria

The eligibility criteria were defined according to the PICOS framework:

- Population: human studies involving healthy or unhealthy individuals of any age.
- Intervention/exposure: ethnic fermented foods Including products that contain naturally occurring or added fermenting microorganisms (e.g., LAB, yeasts, fungi), including EFF in extract or powder form.
- Comparator: control groups with no EFF intake, lower frequency of EFF intake, or intake of corresponding non-fermented products.
- Outcomes: the outcomes were health-related endpoints, as defined by the authors of the included studies. These included clinically relevant effects on gut health, immune modulation, metabolic benefit, cognitive function, cardiovascular, bone, oral, eye and skin health and antimicrobial effects associated with EFF intake. Studies that reported either no effect of EFF or adverse events (e.g., allergic reactions or foodborne outbreaks) were excluded.
- Study design: randomized controlled trials (RCTs), non-randomized controlled studies, uncontrolled trials, and observational studies (cohort, case–control, cross-sectional) were included. Animal and *in vitro* studies and sensorial and analytical evaluations of EFF were excluded.

#### 2.1.4 Data extraction

Data were extracted independently at least by two reviewers using a standardized form. Discrepancies were resolved through discussion and consensus or, if necessary, by consultation with a third reviewer. The following fields were collected: Reference (author and year); Title of the paper; Participants; Country; Study type; Duration of the study; Follow-up time; Type of EFF consumed; Dose/frequency/duration of intervention/exposure; Effect of EFF; Proposed active component/ingredient; Mechanism of action mediated by EFF or by the proposed active component; Control (comparator product; comparator population).

CADIMA software (24) was used to manage the references and to select the studies based on the evaluation, first the titles and abstracts, then full screening of the articles.

### 2.2 Data analysis and synthesis

Due to the heterogeneity in study design, EFF types, and reported clinical outcomes, a narrative synthesis was performed to organize the findings. The synthesis reported:

- Name of EFF;
- Study type;
- Main effect of the EFF;
- Active component causing the health effect;
- Mechanism of action of the active component;
- Origin of the active components (bioactive fraction).

The health-related effects of EFF consumption were synthesized narratively due to heterogeneity in study designs, populations, and outcome measures.

Detailed descriptive summaries were developed for EFF supported by both intervention and observational studies, whereas those based on fewer studies of a single type were only briefly summarized. Visualizations included mapping of EFF and health outcomes and summary tables by region and food group. The synthesis focused on clustering results by shared health indications across these categories. The health-promoting effects of bioactive compounds and their mechanisms of action were documented, with relevant findings sourced from the scientific literature.

Because the included studies were very different in interventions, comparators, outcomes, and reporting formats, and many did not provide effect sizes or measures of variation, we did neither conduct a meta-analysis on, nor grade the evidence for, the health effect of the EFF. Also, the large number of clinical studies identified prevented a systematic analysis of the quality and bias of the individual studies. Instead, although this review was conducted systematically with regard to the study selection, data extraction, and data analysis, a narrative approach was chosen to synthesize the data.

## 3 Results and discussion

A total of 4,566 articles were screened across the three databases. Of these, 1,078 were duplicates and removed, while 3,065 records were excluded based on titles and abstracts. The remaining 423 records were assessed for eligibility based on the full text. The assessment excluded 236 articles. To the remaining 187 articles, 10 were added based on other systematic reviews and after updating the literature search to 2025. From 197 articles, 72 were removed for various reasons (e.g., data were not available or were referring to outbreaks or adverse reactions). The remaining 125 articles were included in the study.

### 3.1 Description of the individual EFF

The results of this systematic narrative review are based on 125 eligible studies reporting changes in clinical endpoints associated with the consumption of EFF. These studies represent a diverse range of food types, geographical origins, and health outcomes. Several studies examined more than one EFF. Also, several foods appeared frequently in the literature, including amazake, boza, buttermilk, injera, kimchi, miso, natto, and tempeh. Most of the eligible human studies were conducted in Asia and Africa, with fewer from Europe and other regions. The focus on these two continents does not result from a bias in the systematic review but, rather, indicates the importance attributed to the production and consumption of local and traditional fermented food in these geographical areas.

#### 3.1.1 Natto (Japan, Asia)

Natto is a traditional Japanese EFF, produced from soybeans that are soaked, steamed or boiled, and fermented using *Bacillus subtilis* var. *natto*. This bacterium is naturally found on rice straw, which was historically used during the fermentation process (25). Natto contains several fermentation-derived compounds, including nattokinase, soy isoflavones, *γ*-polyglutamic acid, and vitamin K2 (26). Thirty-two studies, mostly conducted in Japan, reported changed in clinical endpoints associated with natto consumption.

#### 3.1.2 Miso (Japan, Asia)

Miso is an umbrella term for Japanese seasoning pastes traditionally made from steamed soybeans, barley, or rice. The fermentation process is typically carried out using the koji fungus *Aspergillus oryzae* (27). In addition to its high protein content and soy-derived isoflavones, miso contains minerals such as potassium and sodium in notable amounts (28). Twenty-height studies, mostly conducted in Japan, reported changed in clinical endpoints associated with miso consumption.

#### 3.1.3 Tempeh, tempe, tempeh gembus (Indonesia, Malaysia, Asia)

Tempe, also called tempeh or tempeh gembus, is a product from dehulled boiled soybeans that are fermented by *Rhizopus* spp., namely *R. oligosporus*, *R. oryzae,* and *R. stolonifer*. Tempe has a very compact structure of soybeans that are tightly linked during fermentation by the mycelium of the specific *Rhizopus* strain (29). Twenty-one studies, mostly conducted in Indonesia, reported changes in clinical endpoints associated with Tempe consumption.

#### 3.1.4 Kimchi (South Korea, Asia)

Kimchi is a very popular fermented Korean food, made from Chinese cabbage, other aromatic vegetables, spices, and seasoning. It is produced by LAB. Next to Korea, kimchi is mostly consumed in East Asia. Kimchi is regarded as a healthy food due to its nutritional value and several bioactive compounds (30) and as a potentially probiotic food due to its high content of LAB (31). Seventeen studies reported changes in clinical endpoints associated with Kimchi consumption.

#### 3.1.5 Amazake (Japan, Asia)

Amazake, a very popular drink in Japan, is made of fermented rice. Two different types of amazake beverages which differ in the brewing process: Koji amazake (made from rice-koji) and Sakekasu amazake (made from sake lees) (32). Koji amazake is a non-alcoholic sweet rice beverage, fermented by the koji fungus *Aspergillus flavus* var. *oryzae* and related species (33), leading to degradation of rice starch and formation of glucooligosaccharides (32). Sakekasu amazake is a sweet drink fermented by koji fungi and yeasts, thus containing minor amounts of alcohol, that is made from sake lees dissolved in sugared water (32). Amazake beverages contain nutritional compounds like glucose, amino acids, and B vitamins (34). Seven studies, conducted in Japan, reported changes in clinical endpoints associated with Amazake consumption.

#### 3.1.6 Boza (Turkey)

Boza is a traditional fermented cereal beverage originating from Turkey. It is primarily produced from millet but may also include grains such as barley, bulgur, maize, oats, rice, or wheat. Variants of this beverage are also found in Eastern Europe (known as braga or brascha), the Balkans (busa), Egypt (bouza), Nigeria, and other African countries (bousa or bouza). In preparation, the grains are ground or sifted into flour, then boiled with water, cooled, and strained. Saccharose is added (15–20%), and fermentation is initiated by backslopping with a previous batch or by adding yogurt or sourdough. The process is driven by yeasts and LAB, resulting in a slightly acidic and carbonated beverage. The final product is stored at 4° C (35, 36). Two studies reported changes in clinical endpoints associated with boza consumption.

#### 3.1.7 Injera (Ethiopia)

Injera, also known as enjera, is a traditional Ethiopian pancake-like flatbread made from teff (*Eragrostis tef*) flour. The fermentation process involves a combination of LAB and yeasts. For preparation, milled teff is mixed with water and inoculated with ersho, a starter culture obtained from a previous fermentation of teff flour slurried with water and fermented by naturally occurring microorganisms. After the primary fermentation, a portion of the batter is boiled and recombined with the raw batter. This step is followed by a second, shorter fermentation phase, after which the final batter is baked on a hot surface to form the flatbread. Injera typically has a shelf life of a few days (37, 38). Four studies reported changes in clinical endpoints associated with injera consumption.

#### 3.1.8 Buttermilk (Europe)

Buttermilk is a fermented dairy beverage and a by-product of the dairy industry. The term “buttermilk” encompasses a variety of beverages that differ by production method and regional context. According to the literature, the category includes natural buttermilk, cultured buttermilk, sour milk, cultured milk, cultured skimmed milk, as well as Scandinavian and Bulgarian fermented milk varieties. The most common type is cultured buttermilk, typically produced by fermenting skimmed milk with LAB, whereas whey buttermilk results from the fermentation of cheese whey (39). Buttermilk contains water-soluble milk components, including proteins and phospholipids, along with naturally present or added LAB (40). Six studies reported changes in clinical endpoints associated with buttermilk consumption.

### 3.2 Analysis of EFF by raw material

Beyond the individual food descriptions, the included studies can also be grouped by the primary raw materials used, indicating common health effects across dairy, cereal, soy, and other fermented products.

#### 3.2.1 Dairy products

Fermentation of traditional dairy products is commonly carried out through spontaneous fermentation driven by naturally occurring LAB. In some cases, this process is supported by backslopping or by adding defined starter cultures to guide fermentation more consistently. This review identified 19 studies that reported changes in clinical endpoints associated with the consumption of ethnic fermented dairy products. These include buttermilk (*n* = 6), dahi (*n* = 5), mabisi (*n* = 2), one study each for koumiss, skyr, and one joint study for lben, raib, saykok, and jben.

Dahi is a commonly consumed yogurt in India (41), typically fermented by mixtures of LAB, mainly *Lactococcus lactis* and its subspecies together with *Streptococcus thermophilus*. It is preferred over milk due to several reasons, e.g., sensory and therapeutic benefits, nutritive content, as well as improved shelf life (42).

Mabisi is a traditional Zambian fermented milk product prepared through spontaneous fermentation of raw milk, with multiple regional variations in production methods (43). Lben, raib (also known as rayeb), and jben are Moroccan dairy products derived from cow’s or goat’s milk. Lben is a sour milk drink, raib refers to its gelled form, and jben is a type of soft white cheese (44). Koumiss, also referred to as qymyz or kumis, is a mildly alcoholic and acidic fermented drink made from mare’s milk in Central Asia, produced with LAB and yeasts (45). Skyr is a high-protein Icelandic yogurt made from skimmed milk (46).

#### 3.2.2 Cereal products

This systematic review identified five human studies associating the consumption of ethnic fermented cereal products with changes in clinical endpoints. The EFF investigated included chibwantu and munkoyo (*n* = 2), Acida and Kisra (*n* = 1), Kenkey (*n* = 1), and Togwa (*n* = 1). Chibwantu and munkoyo are two similar Zambian fermented non-alcoholic cereal beverages typically made from maize. The powdered grains are soaked and mixed with roots of several plants to provide amylolytic enzymes to degrade the starch, which is necessary to enable the growth of microorganisms involved in the fermentation process of the filtered liquid (47). Acida and kisra are traditional Sudanese foods made from fermented sorghum or pearl millet, while acida is prepared as porridge and kisra is baked as a flatbread (48, 49). Kenkey is a cooked staple food from Ghana, which is produced by preparing a spontaneously fermented sourdough from maize (50). Togwa is a fermented cereal beverage from Tanzania, made from maize, sorghum, finger millet, or a mixture thereof, produced via spontaneous fermentation by autochthonous microorganisms, e.g., yeasts and LAB (51).

#### 3.2.3 Soy products

In addition to natto, tempeh, and miso, several other ethnic fermented soy-based foods were identified in this review, each described in one human study. These include furu and jang. Furu, also known as sufu, is a Chinese fermented soybean curd made from tofu. Depending on the production method, four main types of sufu are distinguished: Naturally fermented sufu (spontaneous fermentation), mold-fermented sufu (using species of the genera *Actinomucor*, *Mucor*, and *Rhizopus*), bacteria-fermented sufu (using *Bacillus* or *Micrococcus* species), and sufu ripened with enzymes derived from added koji (52).

Jang is an umbrella term for several Korean fermented soybean products, including chungkukjang, kanjang, doenjang, and kochujang (or gochujang). Chungkukjang is comparable to natto and is fermented using *Bacillus subtilis*. For the production of kanjang, doenjang, and kochujang, the intermediate product meju is first prepared by solid-state fermentation of soybean blocks using *Bacillus* spp. and fungi, followed by drying. Kochujang, also known as red pepper paste, is made by mixing ground meju with powdered red pepper, malt-digested rice syrup, rice flour, and salt, then left to ferment. Kanjang and doenjang are produced by immersing meju in brine for liquid fermentation. The supernatant is used to make soy sauce (kanjang), while the solid residue becomes the seasoning paste doenjang (53).

#### 3.2.4 Other fermented food products

This review identified eight human studies reporting changes in clinical endpoints associated with the consumption of EFF made from diverse raw materials. One study focused on both dosa and idli, while another examined both tarhana and boza. The remaining studies addressed dhokla, ufu, poi, and budu, each covered individually.

Dosa, dhokla, and idli are traditional Indian fermented foods made from rice and black gram. Dhokla also contains Bengal gram. The dough is naturally fermented by yeasts and LAB. Following fermentation, idli and dhokla are typically steamed, while dosa is prepared as a thin, crispy pancake (54).

Fufu is a starchy, dough-like food from Nigeria, produced by fermenting cassava tubers with naturally occurring microorganisms. This process helps reduce the content of cyanogenic glucosides found in cassava (55).

Poi is a porridge-like food from Polynesia, made from cooked taro tubers that are mashed, mixed with water, and allowed to undergo spontaneous fermentation, dominated by LAB and yeasts (56).

Tarhana is a traditional fermented food widely consumed in Turkey, prepared from a mixture of wheat flour, yogurt, baker’s yeast, various vegetables, salt, and spices. Fermentation involves *Saccharomyces cerevisiae* and LAB originating mainly from the yogurt. After fermentation, the dough is dried and powdered for later preparation as a soup (57). Similar fermented cereal-dairy products are found in several Central Asian, Southeast European, and Middle Eastern countries, although they differ in ingredients, processing methods, and names.

Budu is a Malaysian fermented fish sauce made from salted anchovies. Once fermentation is complete, it is blended with coconut palm sugar, tamarind, and spices (58).

**Figure 2:**
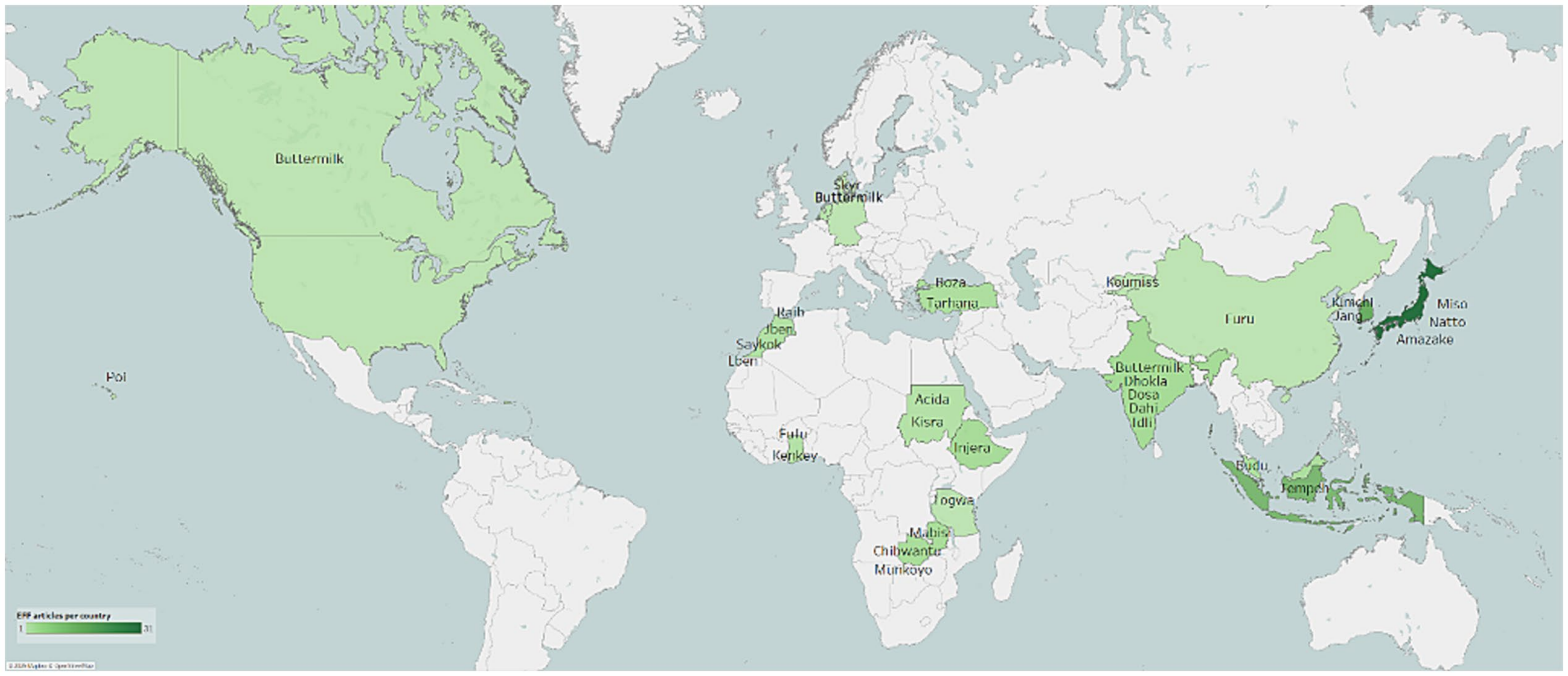
World map highlighting the ethnic fermented foods most frequently mentioned in the 125 reviewed studies, based on their reported country of origin. Map visualisation created with tableau desktop professional edition 2023.3.2 (20233.24.0112.1233). Map background tiles ^©^Mapbox, data ^©^OpenStreetMap contributors.

Figure 2 shows a map, based on the information presented in sections 3.1 and 3.2, highlighting the regions with the most frequently investigated EFF in human studies.

### 3.3 Bioactive compounds activity in EFF

EFF offer a range of potential health benefits for people of all ages, whether they are healthy or have specific diseases. The clinical endpoints changed by the consumption of the EFF as reported in the 125 articles listed in Supplementary Table S2 are summarized in Table 1. The most prominent bioactive components are described in more detail in the following sections.

**Table 1:** Summary of main effects, active components, and mechanisms of action associated with ethnic fermented foods.

EFF	Study type	Main clinical endpoints changed	Active component	Mechanism of action	Bioactive fraction
Amazake	Intervention (RCT)	1. Skin improvement, water loss reduction, sebum reduction; 2. Improve defecation and fecal microbiota composition.	1. GABA, Glucosylceramide. 2. *A. oryzae* cells, glucooligosaccharides (GlcCer), lactic acid.	1. GABA enhances collagen and elastin production, improving skin elasticity. Glucosylceramide contributes to improved skin barrier function and moisture retention; 2. Probiotics and prebiotics improve the metabolism of SCFA that enhances feces production and quality. The GlcCer improves skin barrier function and therefore might affect bowel movements.	Starter culture; fermentation product
Amazake	Observational	1. Reduction of constipation symptoms; 2. Improvement of various symptoms of liver cirrhosis.	1. Isomalto-oligosaccharide; SCFA 2. Amino acids.	1. Isomalto-oligosaccharide promote the growth of *Lactobacillus* and *Bifidobacterium*. The production of SCFA by bacteria promotes intestinal peristalsis and prevents constipation, lowering intestinal pH; 2. BCAA influences the local immune system of the liver and may improve the phagocytic function of neutrophils and NK cell activity of lymphocytes in cirrhotic patients.	Raw matrices; fermentation product
Boza	Intervention (RCT)	Improvement of body fat and bad cholesterol levels. Significant increase in chlorine and creatine levels.	Not determined	Boza consumption decreases triglyceride and VLDL levels. Increased creatine levels in boza are important for continuation of exercise and shortening of recovery time after exercise.	Fermentation product
Boza	Observational (Cross-sectional study)	Decrease of lactational mastitis incidents	LAB	LAB prevent pathogen colonization in mammary glands through antimicrobial and anti-inflammatory effects	Starter culture
Buttermilk (Lassi)	Intervention (RCT)	1. Effects on rehydration, body temperature, heart rate, cortisol levels, kidney function. 2. Reduction of LDL cholesterol and triglyceride levels.	1. Proteins, riboflavin, potassium, vitamin B12, calcium. 2. MFGM components	1. Extra energy and nutrition from buttermilk. 2. MFGM lipids inhibit intestinal cholesterol absorption	Raw matrices
Buttermilk (Lassi)	Observational (Cross-sectional study)	1. Better executive functioning. 2. Improved symptoms of IBD, compared to low-fat yogurt reduced rate of death	1. LAB, bioactive peptides, vitamin K2. 2. Reduced lactose.	1. Impact on gut microbiota to nervous system called gut-brain axis. Probiotics increase tryptophan, which enhances the synthesis of serotonin in the brain. Vitamin K2 is positively associated with cognition. 2. The reduction of lactose in fermented lassi makes it more tolerable for IBD patients. Improved body fat and lipid profile.	Raw matrices; starter culture; fermentation product
Injera	Intervention	Increased satiety and lower glycemic index values	Carbohydrates (incl. starch)	Slower glucose release and absorption in low-GI foods	Raw matrices
Injera	Observational (Cohort study; Case–control study)	1. Increased gut microbiome diversity in infants. 2. Reduced risk of anemia.	1. LAB. 2. Iron, dietary fiber	1. LAB from fermented food promote gut microbial diversity. 2. Increased dietary iron intake and improved bioavailability	Raw matrices; starter culture; fermentation product
Kimchi	Intervention (RCT)	1. Beneficial bacteria decrease the onset and progression of colorectal cancer (CRC); 2. Improvement in IBS symptoms; 3. Improvement in pepsinogen I/II ratio, body weight, BMI, and cholesterol levels, increase in HDL-C and skeletal muscle mass, decrease in serum IL-1β levels, LDL-C, triglycerides, and IL-6, reduced body fat percentage, reduced harmful gut enzymes; 4. Gut microbiota improvement and modulation.	1. Fermented microbiota in kimchi, including *Lactobacillus* spp.; 2. Dietary fiber, LAB; 3. LAB, dietary fiber, bioactive compounds from kimchi ingredients. Phytochemicals from functional kimchi ingredients (e.g., mustard leaves, Chinese pepper, mistletoe, probiotics); 4. LAB, probiotics.	1. Suppression of the CRC-assisted microbiome; 2. Increase of dietary fiber intake, reduction of inflammatory cytokines, modulation of gut microbiota, reduction of harmful fecal enzymes; 3. Improvement of gut microbiota, reduction of pro-inflammatory cytokines, enhancement of lipid metabolism; 4. Gut microbiota modulation. Activity against *H. pylori*. Increase of Bacteroidetes, *Faecalibacterium*, Proteobacteria and Actinobacteria (negative correlation with body fat percentage) and reduction of Firmicutes and *E. coli*. Negative correlation between *B. longum* and waist circumference.	Raw matrices; starter culture; fermentation product
Kimchi	Observational	1. Decrease of colorectal cancer risk; 2. Weight loss and normal weight maintenance; 3. Reduction of total cholesterol, triglycerides, and LDL-C in women; increase of HDL-C in men; 4. Decrease of depressive symptoms in males; 5. Reduction of asthma prevalence. Potential antioxidative, anti-inflammatory effects; 6. Improvement in glucose tolerance, reduction of insulin resistance; 7. Reduction of rhinitis prevalence; 8. Reduction of genetic risk for Aged Related Cataract; 9. Lower prevalence of atopic dermatitis.	1. LAB, carotenoids, vitamins, dietary fiber; 2. LAB, dietary fiber, probiotics, seasoning (garlic, onion and ginger); 3. LAB, seasoning components (garlic, red pepper, ginger); 4. Probiotics; 5. LAB, dietary fiber, phytochemicals; 6. LAB, bioactive compounds; 7–8. LAB; 9. LAB, antioxidants, vitamin B and K;	1. Anticarcinogenic effect of kimchi is linked to increased LAB and high levels of β-carotene, vitamin C, and dietary fiber; 2. Anti-inflammatory properties, cholesterol-lowering activity, probiotic LAB in kimchi regulate adipogenesis-related genes, reducing lipid accumulation and improving metabolism. Garlic, onion and ginger have an anti-obesity effect; 3. LAB contribute to the lipid improvement effect. Garlic lowers cholesterol biosynthesis; capsaicin in red pepper activates PPARα, reducing lipid levels. Ginger is rich in 6-gingerol that reduces TC and TG levels; 4. Probiotics that produce neurotransmitters can significantly influence neural biochemistry, mood, and behavior; 5. Possible immune-modulating effects via gut microbiota and anti-inflammatory properties; 6. Enhanced glucose metabolism, insulin sensitivity improvement; 7. LAB enhance intestinal mucosa immune cells; 8. Gut microbiota enhance lactose and galactose that may mitigate oxidative stress and reduce osmotic pressure in the lens; 9. Probiotics restore balance between the Th1/Th2 cell responses. Vitamins K and B-12 suppress the inflammatory response.	Raw matrices; starter culture; fermentation product
Miso	Intervention (RCT)	1. Intake of miso, despite its high salt content, does not influence daytime BP while it reduces nighttime BP. 2. Miso significantly increases Coenzyme Q10 bioavailability.	1. Inhibitors of Angiotensin Converting Enzyme (ACE); 2. Not specified	1. Inhibition of the renin-angiotensin system and thus promotion of diuresis; 2. Hydrophobic interactions are implied	Raw matrices; starter culture; fermentation product
Miso	Observational	1. Miso soup intake is associated with reduced risk of hypertension as well as improved glycemic control. 2. Lower prevalence of sarcopenia in women. 3. Hormonal Health & reproductive outcomes. 4. Lower risk of inadequate infant sleep duration. 5. Reduced risk of functional constipation. 6. Cancer Risk Reduction. 7. Improved mental health.	1. Isoflavones. 2. omega-3, omega-6. 3. Phytoestrogens. 4. LAB. 5. Dietary fiber. 6. Isoflavones. 7. Proteins	1. A significant effect on the blood pressure has been demonstrated, underlying mechanism is not fully understood. It may involve improvement of autonomic balance. 2. Increased omega-3 and omega-6 intake which is important for muscle maintenance. 3. Modulation of estrogen metabolism. 4. Affects maternal gut microbiota, influencing infant circadian rhythm development. 5. High dietary fiber may improve stool consistency and promote bowel movements. 6. Isoflavones have anticarcinogenic effects. 7. A high protein content is crucial for neurotransmitter synthesis and brain function and is essential for mental health	Raw matrices; starter culture; fermentation product
Natto	Intervention (RCT)	1. Natto intake suppresses postprandial blood glucose levels 2. Natto intake prevents bone loss in postmenopausal women and in aged individuals	1. gamma-polyglutamic acid (γ-PGA) (produced within the fermentation process) 2. Bioavailable isoflavones (in their aglycone form, e.g., Genistein) and/or vitamin K2 (a 40-g pack of natto contains 350 mg of menaquinone 7, a remarkably high amount for a food)	1. Through a mechanism similar to that of β-glucan (delayed gastric emptying and absorption) 2. Isoflavones seem to reduce bone resorption through estrogenic mechanisms	Raw matrices; starter culture; fermentation product
Natto	Observational	1. Diets rich in natto are inversely associated with cardiovascular-mortality risk. 2. Diets rich in natto are associated with decreased bone loss in aging. 3. Natto intake is significantly associated with lower dementia risk. 4. Reduced eczema incidence in infants of mothers with daily natto intake	1. Nattokinase; 2. Isoflavones, vitamin K2. 3. Folates. 4. *B. subtilis* metabolites	1. Reduction in systolic and diastolic BP; Prohibition of blood clots; Reduction of blood glucose levels. 2. Enhanced bone mineralization through vitamins. Isoflavones have a protective effect against bone loss through oestrogenic mechanisms. 3. Neuroprotective effects via anti-inflammatory, anti-oxidant, and homocysteine-lowering pathways. 4. Immune modulation, gut microbiota improvement.	Raw matrices; starter culture; fermentation product
Tempeh	Intervention (RCT)	1. Appetite regulation and nutritional effects; 2. Estrogen regulation; 3. Improvements in global cognitive scores; 4. Improved lipid profiles (LDL, cholesterol, triglycerides); 5. Blood glucose and Insulin regulation; 6. Anti-Inflammatory effects; 7. Calcium bioavailability; 8. Improving maternal iron levels and hemoglobin levels.	1. Bioactive peptides and arginine enhanced by fermentation. High fiber, low GI components; 2. Isoflavones; 3. Isoflavones, probiotics; 4. Dietary fiber, proteins; 5. Fibers; 6. Fiber, UFA (oleic, linoleic, linolenic FA), antioxidants; 7. Calcium, isoflavones; 8. Iron, vitamin C.	1. Fermentation enhances the bioavailability of proteins and arginine, which stimulates insulin release and appetite regulation; 2. Isoflavones mimic estrogen and may exert estrogenic or anti-estrogenic effects. The more available isoflavones might play a role in cancer prevention; 3. Butyrate by probiotics increases the secretion of BDNF; 4. Fibers bind bile acids, reducing cholesterol absorption; 5. Fibers slow glucose absorption, reduce TG levels and improve insulin sensitivity; 6. Anti-inflammatory effects through dietary fiber, improvement in lipid profiles, potential impacts from antioxidants and bioactive compounds; 7. Calcium bioavailability 8. Enhanced iron absorption with reduced phytate effect.	Raw matrices; starter culture; fermentation product
Tempeh	Observational	1. Reduced anemia; 2. Increased weight, height, energy and macronutrient intake; 3. Cognitive recall enhancement; 4. Improved obesity and type-2 diabetes parameters in the human gut.	1. High iron content in soybean; 2. Carbohydrate, protein, vitamin A, zinc, fermented soy protein, bioactive peptides, high fibers; 3. Isoflavones, folate; 4. Soluble fibers, polyphenols, probiotics	1. High fiber amount in tempeh leads to slower Fe absorption and therefore reduced impact on anaemia risk; 2. Carbohydrate and protein content are responsible for weight increase, while vitamin A and zinc increase appetite; 3. Isoflavones may have estrogenic effects, which could support cognitive function in midlife individuals; 4. Gut microbiota modulation, immune function enhancement.	Raw matrices; starter culture; fermentation product
					
Acida	Intervention (RCT)	Most favourable post-prandial glucose and insulin responses	Carbohydrates	Slowly digested carbohydrates result in reduced glycemic and insulin responses	Raw matrices
Budu	Observational (Case–control study)	Protective effect against *H. pylori* infection	LAB	Probiotics from LABmay alter gastric microbiota and suppress *H. pylori*	Starter culture
Chibwantu	Observational (Cross-sectional study)	Contribution to the children’s dietary intake	Microbiota from fermented dairy and cereals, macronutrients, micronutrients	Improved gut microbiota and nutrient bioavailability, potential role in reducing child malnutrition	Raw matrices; starter culture; fermentation product
Dahi	Intervention (RCT)	1. Increased immune cells and their activity. 2. Significant reduction of the duration of diarrhoea. 3. Reduced salivary *S. mutans* count	LAB and proteins	Improved gut and oral microbiota composition leads to better systemic immune responses and reduction of pathogens	Starter culture
Dhokla	Intervention	Improvements in height, weight, skeletal development and biochemical status	Bioactive components, including calcium vitamin A	Nutritional enhancement	Raw matrices
Dosa	Intervention	Lower postprandial glucose levels	Dietary fiber, non-starchy polysaccharides	High dietary fiber slows carbohydrate digestion and glucose absorption. Resistant starch content could contribute to a delayed glycemic response	Raw matrices
Fufu	Intervention (RCT)	Lowered the glycemic index	Dietary fiber	Fiber slows down digestion, which in turn slows down the absorption of glucose into the bloodstream	Ingredients from raw material *it is not clear if fufu was fermented or not
Furu	Intervention (RCT)	In the red furu group serum concentrations of vitamin B12 and folate were negatively associated with homocysteine, and vitamin B-12 was positively associated with folate	Folate, vitamin B12	Folate and vitamin B12 reduce homocysteine, which is associated with chronic diseases, especially vascular diseases	Fermentation product
Idli	Intervention	Delayed peak glucose responses	Low glycemic index carbohydrates	Increased desired glycaemic effect, i.e., delayed peak rise, low glucose response curves	Raw matrices
Jang	Observational (Cohort study)	Inverse association with MetS risk components, including waist circumference and fat mass. Improved serum glucose and HDL cholesterol levels	Bioactive peptides, isoflavones	Anti-inflammatory and lipid-modulating effects	Raw matrices
Jben	Observational (Case–control study)	Jben intake was inversely related to colorectal cancer risk across all locations of the bowel	LAB, calcium, reduction of lactose	LAB establish a barrier preventing the development of pathogens. LAB have anti-inflammatory properties to protect against CRC risk. Lactose association with CRC risk can be reduced through fermentation. Calcium intake has inverse relationship with CRC risk	Starter culture; fermentation product
Kenkey	Intervention (RCT)	Relatively low GI response	Dietary fiber	Slows glucose release and reduces postprandial glycemia	Raw matrices
Kisra	Intervention (RCT)	Lower post-prandial glucose and insulin responses	Carbohydrates	Slowly digested carbohydrates result in reduced glycemic and insulin responses	Raw matrices
Koumiss	Intervention	Reduced cholesterol and triglycerides, increased HDL	LAB, ethanol, carbon dioxide	Fermentation products improving lipid profile	Starter culture; fermentation product
Lben	Observational (Case–control study)	Lben intake was inversely related to colorectal cancer risk across all locations of the bowel	LAB, calcium, reduction of lactose	LAB to establish a barrier preventing the development of pathogens. LAB have anti-inflammatory properties to protect against CRC risk. Lactose association with CRC risk can be reduced through fermentation. Calcium intake has inverse relationship with CRC risk	Starter culture; fermentation product
Mabisi	Observational (Cross-sectional study)	1. Improved Height and Weight for-Age Z-scores (HAZ & WAZ). 2. Sufficiently improve fat, calcium, iron and zinc	Microbiota from fermented dairy and cereals, macronutrients, micronutrients	Improved gut microbiota and nutrient bioavailability, potential role in reducing child malnutrition. Degradation of phytates increases mineral bioavailability	Starter culture; fermentation product
Munkoyo	Observational (Cross-sectional study)	Contribution to children’s dietary intake	Microbiota from fermented dairy and cereals, macronutrients, micronutrients	Improved gut microbiota and nutrient bioavailability, potential role in reducing child malnutrition	Raw matrices; starter culture; fermentation product
Poi	Intervention (RCT)	Significant increases in some probiotic bacteria	LAB	Potential modulation of gut microbiota	Starter culture
Raib	Observational (Case–control study)	Raib consumption was inversely associated with the risk of colorectal and rectal cancer	LAB, calcium, reduction of lactose	LAB establish a barrier preventing the development of pathogens. LAB has anti-inflammatory properties to protect against CRC risk. Lactose association with CRC risk can be reduced through fermentation. Calcium intake has an inverse relationship with CRC risk	Starter culture; fermentation product
Skyr	Intervention (RCT)	Altered abundance of fecal short-chain fatty acid profiles	LAB, SCFA	Improved gut microbiota diversity. Microbial cross-feeding; metabolic alterations	Starter culture; fermentation product
Tarhana	Observational (Cross-sectional study)	Decrease of lactational mastitis incidents	LAB	LAB prevent pathogen colonization in mammary glands through antimicrobial and anti-inflammatory effects	Starter culture
Togwa	Intervention	Significant reduction in enteropathogenic bacteria in fecal swabs. Prevention of diarrhoea	LAB	Gut colonization and inhibition of enteropathogens	Starter culture

Above gray line—the EFF health effect was assessed both in RCTs and observational studies; below gray line—the EFF had indication only in one type of studies. More detailed information can be found in Supplementary Table S2. BCAA, Branched-chain amino acids; FA, fatty acid; IBD, inflammatory bowel disease; CRC, colorectal cancer; LAB, lactic acid bacteria; LDL cholesterol, low density lipoprotein cholesterol; NK cells, natural killer cells; RCT, random controlled trial; SFCA, short chain fatty acids; UFA, unbranched fatty acid; VLDL, very low density lipoprotein; Omega-3, omega-3 fatty acids; Omega-6, omega 6 fatty acid.

Although EFF originate from different regions and are made from various raw materials, recurrent bioactive components can be identified across the 125 studies; these include LAB, isoflavones, dietary fiber, protein, bioactive peptides, amino acids, enzymes, micronutrients (Ca, Fe, Mg, K, Zn), vitamin K1 and K2, other vitamins (A, B, C, B12), glucosylceramide (GlcCer), fatty acids, *B. subtilis*, folate, genistein, polyamines, nattokinase, antioxidants, sphingolipids, oligosaccharide, short chain fatty acids (SCFA), *A. oryzae* cells, GABA, MFGM, phospholipids, carotenoids, ethanol, carbon dioxide, *Subdoligranulum* sp. (from natto), glucosides, and polyphenols.

#### 3.3.1 Lactic acid bacteria

Several interventional and observational studies have highlighted the potential beneficial effects of LAB species across a spectrum of populations and health outcomes, as described below.

##### 3.3.1.1 Gastrointestinal health

LAB are widely recognized for their probiotic properties and various health benefits have been documented across multiple interventional and observational studies involving EFF. A key function of LAB observed in human studies involves modulation of the gut microbiota, in particular the promotion of microbial diversity and an increase in beneficial taxa, including *Bifidobacterium* and *Faecalibacterium* (18, 59, 60). In line with the anti-infectious properties of LAB, frequent consumption of budu, a Malaysian fermented anchovy sauce, is significantly associated with a lower prevalence of *Helicobacter pylori* infection. Alterations in the gastric environment mediated by LAB and other bioactive components in fermented condiments was proposed as a mechanism for this function (61). In children with acute diarrhoea, the consumption of dahi, an Indian fermented milk containing *Lactobacillus acidophilus*, *Lactobacillus rhamnosus*, and *Lactobacillus delbrueckii*, significantly reduced the duration and severity of symptoms. These benefits were linked to gut microbiota modulation and immune response enhancement through increased fecal IgA production (62). Mothers consuming injera, a fermented flatbread made from teff, demonstrated a significant increase in gut microbiome diversity in their infants. This phenomenon may be linked to the transfer of beneficial microbes, particularly LAB like *Lactobacillus* spp., from the maternal diet to the infant’s gut (63). Kimchi, a traditional Korean fermented vegetable food product, has consistently demonstrated effects on various clinical endpoints, primarily due to its high levels of LAB. In a clinical trial, Park et al. (64) found that consuming 100 g of fermented kimchi daily for 10 weeks significantly improved fecal microbiota diversity, boosting beneficial genera such as *Bifidobacterium, Faecalibacterium,* and *Akkermansia*. This suggests that LAB in kimchi helps enhancing the gut microbial balance. In line with these findings, a 12-week randomized controlled trial by Kim et al. (65) demonstrated that a daily intake of 210 g of kimchi improved symptoms of irritable bowel syndrome (IBS), including reduced abdominal pain and bloating, by modulating intestinal inflammation and enhancing consumption of dietary fiber and LAB. Finally, daily intake of koji amazake, a fermented rice beverage, significantly improved defecation frequency and fecal weight in healthy adults. These effects were attributed to the synergistic action of *A. oryzae* cells and gluco-oligosaccharides, which function as probiotics and prebiotics, respectively, to modulate the gut microbiota composition (32).

##### 3.3.1.2 Antimicrobial and anticarcinogenic effects

The intake of Moroccan fermented dairy products such as lben and raib has been linked to reducing the risk of colorectal cancer and LAB was proposed to mediate this effect, in particular through improvement of the gut barrier integrity (66). In line with this report, a case–control study by Oh et al. (67) noted an inverse relationship between the risk of colorectal cancer and kimchi consumption; the authors attributed this protective effect to LAB and other bioactive components. Administration of Kimchi powder to rats injected with a carcinogen concluded to a protective effect of LAB through competition with pathogens, acidification of the intestinal environment, and upregulation of protective genes (68), in line with the chemopreventive potential of probiotics reviewed by Morsli et al. (69).

LAB in fermented beverages have shown significant antimicrobial and gut-modulating effects across diverse populations. The consumption of togwa, a traditional Tanzanian fermented maize product, resulted in a marked decrease in enteropathogenic bacteria among young children, highlighting the role of LAB in gut colonization and competitive inhibition of harmful microbes (70).

##### 3.3.1.3 Metabolic benefits

Ethnic fermented foods are rich in beneficial microbes and other bioactive compounds that have been shown to enhance and support metabolic health. A comprehensive prospective cohort study conducted by Tan et al. (71) with over 58,000 participants revealed a significant link between higher kimchi consumption and reduced body weight and obesity risk. The authors credited these outcomes to the anti-inflammatory effects and gut microbiota-supporting properties of LAB and dietary fibers present in kimchi. Furthermore, kimchi intake significantly modulates bile acid profiles in obese rats, suggesting a potential role for kimchi in regulating host bile acid metabolism (72). As probiotic lactobacilli encode bile salt hydrolase, an enzyme shown to reduce cholesterol levels (73), EFF fermented with LAB may help to prevent hypercholesterolemia.

Moreover, human studies indicates that LAB are key in boosting lipid and glucose metabolism by decreasing LDL cholesterol, increasing HDL cholesterol, improving insulin sensitivity, and reducing fasting blood glucose levels (74–76). Furthermore, LAB enhances the metabolism of nutrients, including lactose and galactose. This mechanism may help to mitigate oxidative stress and lower the risk of developing conditions as observed for age-related cataracts in a Korean cohort (77). Regular consumption of koumiss, a fermented mare’s milk, has been associated with improved lipid profiles, including reduced cholesterol and triglyceride levels and increased HDL, likely due to the combined action of LAB and other fermentation products on lipid metabolism (78). Metabolic benefits of miso consumption have primarily been attributed to compounds like isoflavones (79), which are suggested to inhibited the accumulation of visceral fat for diabetics. A recent meta-analysis in patients with diabetes conclude that also the LAB has modulating effect on lipid levels and improves metabolic markers (80).

##### 3.3.1.4 Immune modulation

Expanding on the immunomodulatory effects, a study with dahi, a fermented dairy from India, demonstrated that regular consumption of curd in malnourished children improved both pro-inflammatory (TNF-*α*, IFN-*γ*) and anti-inflammatory (IL-4, IL-10) cytokine levels, indicating a more balanced and functional immune response during nutritional rehabilitation (81). Highlighting the immunomodulatory potential of fermented soy, the prevalence of rhinitis decreased with increasing kimchi consumption (82). This effect is attributed to LAB isolated from kimchi, which are helpful in development and maintenance of the immune system by enhancing intestinal mucosa immune cells (83).

LAB also exhibits anti-inflammatory properties, which may contribute to improve metabolic health, e.g., by managing body weight in overweight and obese patients (84) and immunity, e.g., by being associated with a lower occurrence of asthma in a Korean cohort, probably due to their influence on the gut-immune axis (85).

##### 3.3.1.5 Cognitive function

Enhanced executive functioning in older Dutch adults correlated with a greater consumption of fermented dairy, particularly buttermilk. The cognitive improvements of older adults in a Dutch cohort were attributed to fermented dairy products, presumably through influence of the probiotic activity of the LAB present in the fermented products, as well as other bioactive compounds, on the gut microbiota an the gut-brain axis (86). Tempeh consumption improved the global cognitive scores of adults aged 60 years or over with mild cognitive impairment after 6 months of intervention together with improved language function. The positive effects of Tempeh were attributed to the ability of LAB to activate a signalling process involving the increase in butyrate and subsequently brain-derived neurotrophic factor (BDNF) to finally regulate amyloid beta (Aβ), which promotes neuron damage in Alzheimer-related processes (87).

Collectively, these mechanisms highlight the functional role of LAB as potent bioactive agents with potential benefits for gastrointestinal, antimicrobial, metabolic, immune and cognitive health.

#### 3.3.2 Isoflavones

Isoflavones are a group of bioactive phytoestrogens primarily found in fermented soy foods like miso and natto. Interventional, cohort, and cross-sectional studies have shown a range of health benefits associated with these compounds. Isoflavones influence the body systemically through estrogen receptor modulation, antioxidant effects, and anti-inflammatory processes. Regular consumption of fermented soy products like miso and natto is consistently linked to reductions in visceral fat, better insulin sensitivity, low prevalence of sarcopenia and decreased risks of type 2 diabetes and cardiovascular events (88–91). Additionally, isoflavones play a role in reproductive and prenatal health, with higher miso consumption associated with a lower risk of early preterm birth, likely due to improved immune regulation and greater gut microbiota diversity (92). Their protective effects also extend to cancer, where regular intake has been inversely correlated with all cause mortality (93) and even site-specific cancer mortality, including stomach cancer (94).

Long-term consumption of isoflavones from miso and natto has been associated with neurological benefits such as enhanced cognitive function and a lowered risk of dementia, likely due to their influence on cerebral blood flow and neuroinflammation (87, 95, 96). In addition, isoflavones originating from natto may aid in bone health and alleviate menopausal symptoms (97, 98) by mimicking estrogenic functions, which can be advantageous in states of estrogen deficiency. The protective effects on cardiovascular health are further supported by evidence showing a reduced risk of coronary heart disease and stroke mortality among populations with high soy intake (90, 99). Diets rich in fermented soy are providing the isoflavones responsible of enhancing antioxidant levels and regulate anti-inflammatory cytokine profiles in a variety of populations, including cancer survivors (100). Among the frequently noted advantages are increased metabolic function, mineral bioavailability and better lipid profiles (99, 101, 102). Overall, these human studies emphasize the contribution of isoflavones from ethnic fermented soy foods in promoting metabolic, cardiovascular, immunoregulating and cognitive health across different demographic groups.

#### 3.3.3 Dietary fiber

Dietary fiber, especially from fermented and traditional plant-based foods, is noted for its positive impact on metabolic regulation, gut health, and the prevention of chronic diseases. Numerous studies highlight the blood sugar-lowering benefits of fiber-rich staples like dosa, fufu, and kenkey. In patients with type 2 diabetes, consumption of millet-based dosa led to significantly lower postprandial glucose levels compared to rice-based alternatives, likely due to the high content of non-starchy polysaccharides and slower carbohydrate digestion (103). Similarly, various fufu formulations demonstrated lower glycemic responses in healthy individuals, highlighting the known role of dietary fiber in regulating glucose absorption (104). Low glycemic responses were also recorded following kenkey intake, which is consistent with the described metabolic effects of slowly digestible carbohydrates and dietary fiber (105). In the context of micronutrient absorption and women’s health, higher teff-based injera consumption was associated with reduced anemia prevalence among Ethiopian pregnant women (106), a benefit attributed to both its dietary iron and fiber-enhanced bioavailability. Additionally, fiber-rich fermented vegetables like kimchi have been associated with a lower risk of colorectal cancer in Koreans (67), potentially due to the combined anticarcinogenic effect of fiber and LAB. Dietary fiber can reduce intestinal transit time and dilute potential carcinogens within the gut. Through anaerobic fermentation by gut microbiota, fiber is converted into SCFAs, which stimulate the release of hormones such as GLP-1 and PYY—supporting glucose metabolism by enhancing insulin secretion and regulating blood sugar levels—thus contributing to cancer prevention in obese rats (72).

Recent studies reinforce these findings, indicating that the consumption of fiber-rich fermented foods like kimchi positively impacts gut health, metabolic markers, and immune function. Frequent kimchi intake has been associated with weight loss, better lipid profiles, and a lower occurrence of metabolic and inflammatory diseases such as irritable bowel syndrome and asthma. These benefits are primarily attributed to dietary fiber, LAB, and bioactive phytochemicals (65, 71, 85, 107). In older adults, a high fiber intake from fermented soybean products like miso has been associated with improved psychological well-being and enhanced cognitive outcomes (108), suggesting further neuromodulatory effects of fiber within fermented matrices.

Additionally, studies from Southeast Asia and Europe have reported similar metabolic benefits associated with the consumption of fiber present in EFF. In particular, tempeh, improves postprandial glucose and insulin in healthy humans (109), glucose and insulin in women with hyperlipidemia (110), as well as glucose, insulin, triglyceride, HDL cholesterol, and CRP in obese women (111–113). These findings collectively highlight the significant role of dietary fiber in preventing chronic diseases and maintaining metabolic balance, especially when it is part of culturally relevant, minimally processed, and fermented diets.

#### 3.3.4 Protein-based components

Extensive research has investigated the physiological advantages of protein-based components. Soybean-based fermented foods, which are rich in carbohydrates, proteins, vitamin A, and zinc, led to notable body weight gain in children (114). Fermented soy proteins and bioactive peptides have also been linked to increased energy intake and better nutritional status in underweight children (115). Fermented soy products, particularly miso, have demonstrated notable effects on managing blood pressure (116, 117). Miso contains natriuretic peptides and ACE-inhibitory peptides that lower nighttime blood pressure and arterial stiffness though vascular relaxation and natriuresis (118). Moreover, bioactive peptides and isoflavones found in fermented soy products show an inverse relationship with components of the metabolic syndrome, acting through anti-inflammatory and lipid-modulating mechanisms (119). Children who consumed tempeh showed improvements in both weight and hemoglobin levels due to enhanced protein bioavailability (120). Furthermore, in another study, supplementation with tempeh resulted in significant gains in both weight and height among children suffering from stunting and wasting (121). Fermented miso has also been associated with gastrointestinal benefits (122). As a possible mechanism, these authors discuss the role of amino acids like histidine and glutamate in alleviating reflux and dyspepsia by buffering stomach acid and promoting mucosal protection. Similarly, fermented dairy products, which are abundant in proteins, vitamins, fiber, and minerals, have been linked to enhancements in mental and emotional well-being, possibly through the mechanisms of the gut-brain axis (108). In elderly, buttermilk intake has been shown to improve executive functioning, which is likely due to the presence of bioactive peptides and vitamin K2 (86).

In metabolic and hepatic contexts, the fermented rice drink amazake has been found to alleviate symptoms of liver cirrhosis (123), primarily through the effects of amino acids, glucose, and vitamins that enhance energy metabolism. This beverage has also been found to improve skin hydration and diminish sebum levels, probably owing to fermentation-derived amino acids and sugars that help strengthen the skin barrier (34).

#### 3.3.5 Micronutrients

The micronutrients in EFF significantly enhance health outcomes. For instance, potassium, a regulator of electrolyte balance, may contribute to the beneficial effects of buttermilk on the hydration and thermoregulation of humans placed in a hot environment (124).

Micronutrient-rich EFF also positively influence growth and skeletal development. A classical trial demonstrated that children who consumed fermented cereal-based weaning foods like dhokla fortified with lime-derived calcium increased height and weight and bone growth (125). In the intervention study of Hajare et al. (126) in malnourished children fed dahi, the observed increase in immune cell counts (monocytes, neutrophils, basophils, lymphocytes) may be mediated by trace minerals, such as zinc and selenium, often present in this EFF. Finally, milk fat globule membrane (MFGM) and sphingolipids may contribute to the lowering of cholesterol and triglyceride levels observed in men and women (17).

Of note, although some studies emphasized the role of carbohydrates or the microbial content in mediating the bioactive properties of EFF, the role of micronutrients might have been under-evaluated. For instance, millet porridges consumed in Sudan are primarily rich in carbohydrates but also serve as natural sources of magnesium and potassium (127), potentially contributing to the control of glucose and insulin homeostasis (128).

#### 3.3.6 Vitamin K2

A key component of fermented foods is vitamin K, particularly in the form of menaquinones (vitamin K2 or MK-7), which are produced during bacterial fermentation. Vitamin K2 has been shown to support cognitive health, bone mineralization, and dental preservation. The consumption of vitamin K2-enriched buttermilk was associated with enhanced executive cognitive functioning, likely due to its effect on cerebral blood flow and neurotransmitter synthesis (86). A more exhaustive population-based study validated that vitamin K and B-complex vitamins derived from miso helped alleviate psychological symptoms, emphasizing the vital role of vitamin K2 in mental health and emotional regulation (108).

Consumption of natto by healthy Japanese men (129) markedly raised serum menaquinone-7 (MK-7) but also *γ*-carboxylated osteocalcin levels, which are indicators of bone formation and mineral retention. The benefits of natto for bone health have notably been mostly documented in postmenopausal Japanese women. Katsuyama et al. (130) showed that consumption of natto increased bone formation marker levels; MK-7 supplementation through fermented foods significantly reduce the risk of osteoporotic fractures, primarily by improving bone mineral density and activating osteocalcin (131); consuming natto has also been linked to elevated serum levels of MK-4 and correction of subclinical vitamin K deficiency in bone, underscoring the osteoprotective potential of fermented soy products (132); a higher dietary intake of vitamin K2 (MK-7) has been positively associated with reduced lumbar spine bone loss, likely due to its role as a cofactor in the γ-carboxylation of osteocalcin (97); finally, the intake of fermented foods rich in vitamin K2 and isoflavones has been linked to reduced tooth loss, likely due to improved bone metabolism and the preservation of dental tissues (133).

**Figure 3:**
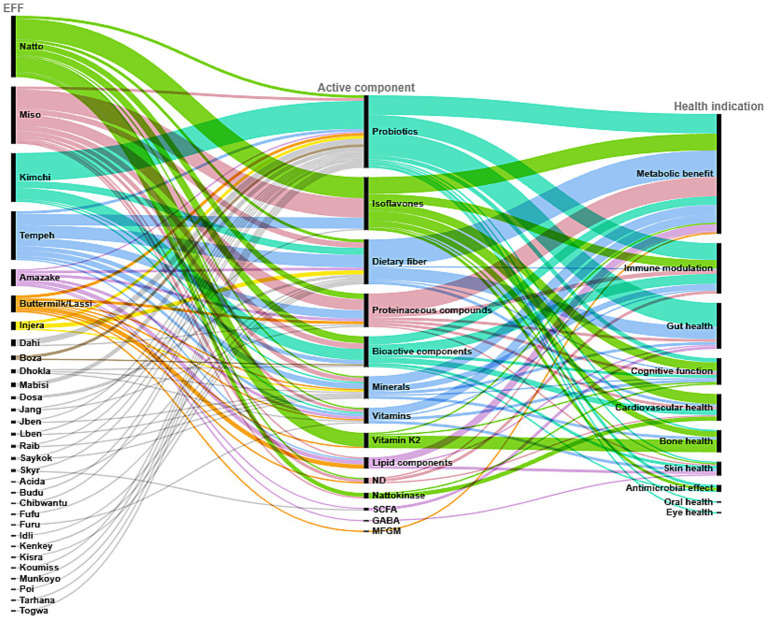
Alluvial diagram highlighting the correlation among EFF, active components, and health outcomes reported in Supplementary Table S2. Lipid components include glucosylceramide, omega fatty acids, sphingolipids, and phospholipids; vitamins (B, A, K, C); proteinaceous compounds include proteins, peptides, and enzymes; GABA (gamma-aminobutyric acid); SCFA (short-chain fatty acid); probiotics include LAB and starter cultures; bioactive components include polyphenols, antioxidants, etc.; minerals (K, Ca, Fe, Zn, Na, Cl, Mg); MFGM (milk fat globule membrane components); ND (not determined). Color coding is based on the list of EFF in Table 1, where the first height EFF are attributed different colors, whereas the remaining are labeled with grey color.

Collectively, these findings highlight the crucial role of bioactive components found in EFF. These fermented foods serve as vehicles for supplying nutrients and compounds enhanced by fermentation, which act together to promote cardiometabolic, immune, gastrointestinal, neurocognitive, osteoprotective and developmental health. Therefore, EFF have a great potential for prevention in public health as well as part of clinical dietary interventions (see Figure 3).

**Figure 4:**
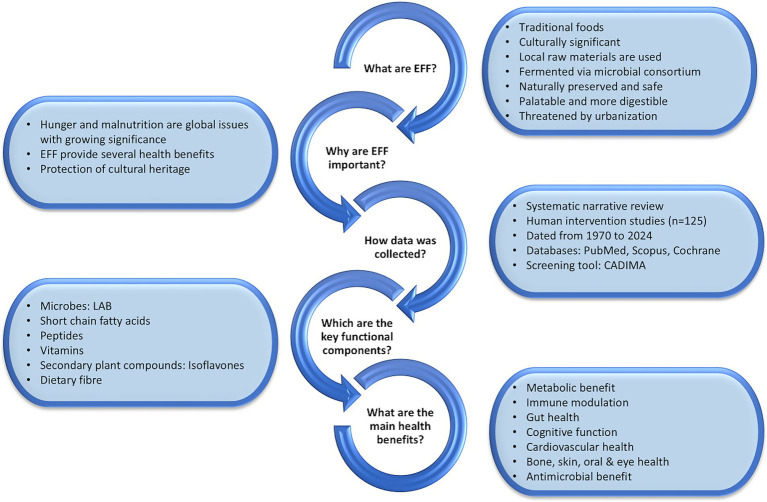
Visual summary of EFF and health outcomes.

To provide an integrated view of these insights, Figure 4 presents a visual summary of the review’s main elements, including the methodology, key functional components, observed health benefits, and concluding perspectives.

### 3.4 Strengths and limitations of the present study

This systematic narrative review gives a comprehensive overview of human intervention and cohort studies examining the health benefits of EFF worldwide. A major strength of this review is that it analyzes data from a large number of articles published from 1970 to 2024 to evaluate evidence supporting the health benefits of EFF. An extensive list of keywords was created based on the literature of Tamang and Thapa (7) and Perricone (8). The list of EFF includes the food items from several regions as well as the raw materials ranging from dairy, fish to meat, legumes to cereals, fruits and vegetables. The primary focus was on the health indications associated with these EFF. Data extraction aimed at finding information about the active components and associated mechanism of action responsible for the observed health benefit. Furthermore, this review discusses whether the reported health benefits of EFF originate from (i) the components already present in the raw material, (ii) the starter cultures used for the fermentation process, or (iii) components derived from the fermentation process. Among these, the contributions from the fermentation process itself may be the most critical, as it can release or transform the bioactive compounds that are otherwise inaccessible in the raw material or absent from starter cultures alone. This highlights the unique role of fermentation in generating health-relevant components that would not emerge without this biochemical transformation.

This systematic narrative review has limitations.

During data extraction, only beneficial outcomes were extracted. These beneficial outcomes are indicated in Supplementary Table S2 and summarized in Table 1. Studies that described neutral or detrimental effects of EFF, including allergic reactions and disease outbreaks related to the studied foods, were excluded (53, 134, 135). While detrimental outcomes are important to report, most of these were often linked to excessive salt content or pre-existing contamination of raw materials (136–138) making those studies less relevant. On the other hand, this systematic review took the decision of only presenting the beneficial outcomes reported by the identified studies to provide a first catalogue of the potential health benefits of EFF. This strategy consequently strongly biased the review towards the beneficial side of the functionality of EFF. The high number of human studies (*n* = 125) reviewed precluded an analysis of their risk of bias. On the same line, the large scope of the review addressed through the research question and the selection of the PICO criteria was not compatible with a meta-analysis of the data. Instead, a narrative approach was taken while keeping the methodology for the review systematic. This methodology allowed to focus on a description of the most often reported EFF and clinical endpoints to highlight the contribution of this diverse category of foods to healthy dietary patterns. However, a critical quantitative appraisal of the quality of the studies as well as of the scientific evidence for the reported effects should be undertaken in future reviews on subsets of the research question addressed here.

The exclusion of non-English publications and the lack of searches in databases such as Embase may have resulted in the omission of relevant studies. Furthermore, the formulation of PICO elements set further constraints for the selection process so that the identification of all relevant studies cannot be claimed. In this review, no intervention or cohort studies from South America or Australia met the eligibility criteria, although Tamang and Thapa (7) have reported EFF from these regions. During data assessment only 2 additional studies were identified through the analysis of other reviews on this topic, indicating that the identification rate of the review is indeed satisfactory. On the other hand, the PICO frame was broadly defined by including all human groups from infants to elderly people, healthy or sick, together with a broad range of EFF listed in the search terms. This resulted in a large number of hits and a vast majority of the articles were excluded upon evaluation of the titles/abstracts and full texts. Notably, although Yakult was captured by the search strategy, human studies investigating this product were finally not considered as it is a globally commercialized probiotic dairy drink produced with standardized starter cultures.

Most of the included studies were conducted in Asia and Africa an only few on populations in other continents thus limiting representativeness of the data at a global level. As this systematic review included only studies published in English a selection bias based on the language of the reported studies might have been introduced.

## 4 Conclusion

This systematic narrative review highlights the consistent health benefits of EFF reported in 125 human studies across diverse regions over five decades. EFF- ranging from cereal-based foods like dosa and injera to soy-based tempeh and dairy products like dahi are associated with improved metabolic regulation, cardiovascular and gut health, cognitive function, and immune response. These benefits arise not only from raw ingredients or probiotic content, but from the fermentation process itself, which enhances nutrient bioavailability, reduces antinutrients, and generates bioactive compounds such as amino acids, peptides, and vitamins (K2, B12, riboflavin, folate). The review emphasizes that fermentation transforms the food matrix in ways that isolated probiotics or raw foods cannot replicate. The review underscores the public health potential of EFF as accessible, culturally rooted, and nutritionally adequate components of human diet.

Preserving traditional fermentation practices while ensuring safety and consistency will be key to advancing their contribution to public health. In addition, as diet-related chronic diseases rise globally, integrating EFF into dietary guidelines could promote sustainable health strategies. The information presented in this review also provides a basis for the food industry to develop culturally authentic, healthy, innovative fermented food products, potentially enabling health claims and broadening the market of functional foods. To unlock the full potential of EFF future research should prioritize clinical trials with products and populations from under-represented geographical areas, investigate mechanisms of action responsible for the observed effects, and contextualize the lessons learned from these studies in the cultural context of their consumption.

## Data Availability

The original contributions presented in the study are included in the article/Supplementary material, further inquiries can be directed to the corresponding authors.
