# Supplementary material for: Health benefits of ethnic fermented foods
Source: Front Nutr. 2025 Sep 26;12:1677478. doi: 10.3389/fnut.2025.1677478 (PMC12512351; doi:10.3389/fnut.2025.1677478)
Supplement: Supplementary file 1 [file Table_1.DOCX]

Supplementary Table S1. Search strategy for bibliographic search of published human studies related to the health benefits and risks of Ethnic Fermented Foods. The “OR” operator separates different food groups.

| **Search string #** | **Search code** |
| --- | --- |
| **PubMed** | |
| #1 | "Atole"[Title/Abstract] OR "Banku"[Title/Abstract] OR "Bhattejaanr"[Title/Abstract] OR "Burukutu"[Title/Abstract] OR "Jinhua" "Skyr"[Title/Abstract] OR "Kaymak"[Title/Abstract] OR "Kumis"[Title/Abstract] OR "Koumiss"[Title/Abstract] OR "Ambra di Talamello"[Title/Abstract] OR "Shanklish"[Title/Abstract] OR "Kajmak"[Title/Abstract] OR "Ayran"[Title/Abstract] OR "Doogh"[Title/Abstract] OR "Buttermilk"[Title/Abstract] OR "Amasi"[Title/Abstract] OR "Filmjölk"[Title/Abstract] OR "Matzoon"[Title/Abstract] OR "Matsoni"[Title/Abstract] OR "Mursik"[Title/Abstract] OR "Viili"[Title/Abstract] OR "Malai"[Title/Abstract] OR "Leben"[Title/Abstract] OR "Yakult"[Title/Abstract] OR "Lassi"[Title/Abstract] OR "Calpis"[Title/Abstract] OR "Chal"[Title/Abstract] OR "Airag"[Title/Abstract] OR "Chhurpi"[Title/Abstract] OR "Dahi"[Title/Abstract] OR "Dadih"[Title/Abstract] OR "Laban rayeb"[Title/Abstract] OR "Lben"[Title/Abstract] OR "Misti dahi"[Title/Abstract] OR "Mishti doi"[Title/Abstract] OR "Lal dahi"[Title/Abstract] OR "Payodhi"[Title/Abstract] OR "Nunu"[Title/Abstract] OR "Philu"[Title/Abstract] OR "Shrikhand"[Title/Abstract] OR "Somar"[Title/Abstract] OR "Sua chua"[Title/Abstract] OR "Tarag"[Title/Abstract] OR "Borhani"[Title/Abstract] OR "Bagoong"[Title/Abstract] OR "Dayok"[Title/Abstract] OR "Ganjang"[Title/Abstract] OR "Garum"[Title/Abstract] OR "Burong Hipon Tagbilao"[Title/Abstract] OR "Jeotgal"[Title/Abstract] OR "Alheira"[Title/Abstract] OR "Androlla"[Title/Abstract] OR "Arjia"[Title/Abstract] OR "Chartayshya"[Title/Abstract] OR "Kargyong"[Title/Abstract] OR "Nham"[Title/Abstract] OR "Musom"[Title/Abstract] OR "Pastirma"[Title/Abstract] OR "Sai Krok Prew"[Title/Abstract] OR "Sai krok Isan"[Title/Abstract] OR "Salchichon"[Title/Abstract] OR "Salsiccia"[Title/Abstract] OR "Soppressata"[Title/Abstract] OR "Sucuk"[Title/Abstract] OR "Suka ko masu"[Title/Abstract] OR "Tocino"[Title/Abstract] OR "Belacan"[Title/Abstract] OR "Blacan"[Title/Abstract] OR "Bakasang"[Title/Abstract] OR "Burong Bangus"[Title/Abstract] OR "Burong Isda"[Title/Abstract] OR "Budu"[Title/Abstract] OR "Gnuchi"[Title/Abstract] OR "Gulbi"[Title/Abstract] OR "Hentak"[Title/Abstract] OR "Hoi-malaeng pu-dong"[Title/Abstract] OR "Jeotgal"[Title/Abstract] OR "Saeoo Jeot"[Title/Abstract] OR "Karati"[Title/Abstract] OR "Bordia"[Title/Abstract] OR "Lashim"[Title/Abstract] OR "Kusaya"[Title/Abstract] OR "Myeolchi jeot"[Title/Abstract] OR "Narezushi"[Title/Abstract] OR "Nam pla"[Title/Abstract] OR "Ngari"[Title/Abstract] OR "Nuoc mam"[Title/Abstract] OR "Patis"[Title/Abstract] OR "Pla paeng Daeng"[Title/Abstract] OR "Plaa Som"[Title/Abstract] OR "Pla khao sug"[Title/Abstract] OR "Shidal"[Title/Abstract] OR "Shottsuru"[Title/Abstract] OR "Sik hae"[Title/Abstract] OR "Suka ko maacha"[Title/Abstract] OR "Sukuti"[Title/Abstract] OR "Surströmming"[Title/Abstract] OR "Tungtap"[Title/Abstract] OR "Caper"[Title/Abstract] OR "Yongfeng chili sauce"[Title/Abstract] OR "Poi"[Title/Abstract] OR "Pao cai"[Title/Abstract] OR "Burong Mustasa"[Title/Abstract] OR "Dhamuoi"[Title/Abstract] OR "Ekung"[Title/Abstract] OR "Eup"[Title/Abstract] OR "Goyang"[Title/Abstract] OR "Gundruk"[Title/Abstract] OR "Khalpi"[Title/Abstract] OR "Mesu"[Title/Abstract] OR "Oiji"[Title/Abstract] OR "Naw Mai Dong"[Title/Abstract] OR "Pak Gad Dong"[Title/Abstract] OR "Sayur asin"[Title/Abstract] OR "Soibum"[Title/Abstract] OR "Soidon"[Title/Abstract] OR "Sinki"[Title/Abstract] OR "Sunki"[Title/Abstract] OR "Takuan"[Title/Abstract] OR "Tuaithur"[Title/Abstract] OR "Bikalga"[Title/Abstract] OR "Masaura"[Title/Abstract] OR "Masyaura"[Title/Abstract] OR "Chikwangue"[Title/Abstract] OR "Fufu"[Title/Abstract] OR "Gari"[Title/Abstract] OR "Lafun"[Title/Abstract] OR "Konkonte"[Title/Abstract] OR "Cheonggukjang"[Title/Abstract] OR "Doenjang"[Title/Abstract] OR "Doubanjiang"[Title/Abstract] OR "Douchi"[Title/Abstract] OR "Pon ye gyi"[Title/Abstract] OR "Ssamjang"[Title/Abstract] OR "Tương"[Title/Abstract] OR "Tungrymbai"[Title/Abstract] OR "Huáng Jiàng"[Title/Abstract] OR "Gochujang"[Title/Abstract] OR "Iru"[Title/Abstract] OR "Sumbala"[Title/Abstract] OR "Ogiri"[Title/Abstract] OR "Ogili"[Title/Abstract] OR "Oncom"[Title/Abstract] OR "Dhokla"[Title/Abstract] OR "Lufu"[Title/Abstract] OR "Jang"[Title/Abstract] OR "Bekang"[Title/Abstract] OR "Chungkokjang"[Title/Abstract] OR "Jeonkukjang"[Title/Abstract] OR "Dawadawa"[Title/Abstract] OR "Furu"[Title/Abstract] OR "Sufu"[Title/Abstract] OR "Hawaijar"[Title/Abstract] OR "Kanjang"[Title/Abstract] OR "Kawal"[Title/Abstract] OR "Kecap"[Title/Abstract] OR "Ketjap"[Title/Abstract] OR "Kinema"[Title/Abstract] OR "Meitauza"[Title/Abstract] OR "Meju"[Title/Abstract] OR "Oncom"[Title/Abstract] OR "Okpehe"[Title/Abstract] OR "Soumbala"[Title/Abstract] OR "Shoyu"[Title/Abstract] OR "Tauco"[Title/Abstract] OR "Thua nao"[Title/Abstract] OR "Ugba"[Title/Abstract] OR "Wari"[Title/Abstract] OR "Yandou"[Title/Abstract] OR "Trachana"[Title/Abstract] OR "Tarhana"[Title/Abstract] OR "Eftazimo"[Title/Abstract] OR "Tianmianjiang"[Title/Abstract] OR "Murri"[Title/Abstract] OR "Appam"[Title/Abstract] OR "Chakuli pitha"[Title/Abstract] OR "Dosa"[Title/Abstract] OR "Enduri Pitha"[Title/Abstract] OR "Idli"[Title/Abstract] OR "Injera"[Title/Abstract] OR "Enjera"[Title/Abstract] OR "Injera Gowé"[Title/Abstract] OR "Kenkey"[Title/Abstract] OR "Khanom chin"[Title/Abstract] OR "Kuzhi paniyaram"[Title/Abstract] OR "Mixian"[Title/Abstract] OR "Ogi"[Title/Abstract] OR "Akamu"[Title/Abstract] OR "Idly"[Title/Abstract] OR "Amazake"[Title/Abstract] OR "Chicha"[Title/Abstract] OR "Boza"[Title/Abstract] OR "Bushera"[Title/Abstract] OR "Busa"[Title/Abstract] OR "Ben saalga"[Title/Abstract] OR "Hussuwa"[Title/Abstract] OR "Jalebi"[Title/Abstract] OR "Khamak"[Title/Abstract] OR "Kao mak"[Title/Abstract] OR "Kunu zaki"[Title/Abstract] OR "Kisra"[Title/Abstract] OR "Koko"[Title/Abstract] OR "Mbege"[Title/Abstract] OR "Pito"[Title/Abstract] OR "Poto poto"[Title/Abstract] OR "Puto"[Title/Abstract] OR "Selroti"[Title/Abstract] OR "Sel Roti"[Title/Abstract] OR "Ketan"[Title/Abstract] OR "Togwa"[Title/Abstract] OR "Uji"[Title/Abstract] OR "Tapai"[Title/Abstract] OR "Tapay"[Title/Abstract] OR "Bánh cuốn"[Title/Abstract] OR "Pozol"[Title/Abstract] OR "Rabadi"[Title/Abstract] OR "Kimchi"[Title/Abstract] OR "Tempe"[Title/Abstract] OR "Miso"[Title/Abstract] OR "Natto"[Title/Abstract] OR "Amabere"[Title/Abstract] OR "Amaruranu"[Title/Abstract] OR "Ergo"[Title/Abstract] OR "Fènè"[Title/Abstract] OR "Fene"[Title/Abstract] OR "Gariss"[Title/Abstract] OR "Kule Naoto"[Title/Abstract] OR "Mabisi"[Title/Abstract] OR "Mafi"[Title/Abstract] OR "Masai"[Title/Abstract] OR "Mutandabota"[Title/Abstract] OR "Omashikwa"[Title/Abstract] OR "Pendidam"[Title/Abstract] OR "Nyarmie"[Title/Abstract] OR "Sethemi"[Title/Abstract] OR "Suusac"[Title/Abstract] OR "Zabady"[Title/Abstract] OR "Ben-saalga"[Title/Abstract] OR "Gowé"[Title/Abstract] OR "Kunu-zaki"[Title/Abstract] OR "Mawè"[Title/Abstract] OR "Basterma"[Title/Abstract] OR "Basturma Gueddid"[Title/Abstract] OR "Khlii"[Title/Abstract] OR "Khlia"[Title/Abstract] OR "Msrana"[Title/Abstract] OR "Merguez"[Title/Abstract] OR "Naqaneq"[Title/Abstract] OR "Pastrami"[Title/Abstract] OR "Sujuk"[Title/Abstract] OR "Soudjouk"[Title/Abstract] OR "Feseekh"[Title/Abstract] OR "Momone"[Title/Abstract] OR "Kinda"[Title/Abstract] OR "Cingwada"[Title/Abstract] OR "Bantu beer"[Title/Abstract] OR "Bouza"[Title/Abstract] OR "Bussa"[Title/Abstract] OR "Kachasu"[Title/Abstract] OR "Mangisi"[Title/Abstract] OR "Merrisa"[Title/Abstract] OR "Sifanu"[Title/Abstract] OR "Tchoukoutou"[Title/Abstract] OR "Chhu"[Title/Abstract] OR "Churkam"[Title/Abstract] OR "Kalari"[Title/Abstract] OR "Mar"[Title/Abstract] OR "Mohi"[Title/Abstract] OR "Phiu"[Title/Abstract] OR "Ang-kak"[Title/Abstract] OR "Jalabi"[Title/Abstract] OR "Lao-chao"[Title/Abstract] OR "Nan"[Title/Abstract] OR "Tape Ketan"[Title/Abstract] OR "Khyopeh"[Title/Abstract] OR "Nem-chua"[Title/Abstract] OR "Sai-krok-prieo"[Title/Abstract] OR "Sa-um"[Title/Abstract] OR "Satchu"[Title/Abstract] OR "Tocin"[Title/Abstract] OR "Balao-Balao"[Title/Abstract] OR "Hoi-Malaeng"[Title/Abstract] OR "Ika-Shiokara"[Title/Abstract] OR "Jeotkal"[Title/Abstract] OR "Myulchijeot"[Title/Abstract] OR "Nampla-Dee"[Title/Abstract] OR "Nampla-Sod"[Title/Abstract] OR "Pla-Paeng-Daeng"[Title/Abstract] OR "Pla-Som"[Title/Abstract] OR "Pla-Khao-Sug"[Title/Abstract] OR "Pu-Dong"[Title/Abstract] OR "Sheedal"[Title/Abstract] OR "Sidra"[Title/Abstract] OR "Sikhae"[Title/Abstract] OR "Bhallae"[Title/Abstract] OR "Maseura"[Title/Abstract] OR "Oncom Hitam"[Title/Abstract] OR "Oncom Merah"[Title/Abstract] OR "Papad"[Title/Abstract] OR "Axone"[Title/Abstract] OR "Aakhoni"[Title/Abstract] OR "Grep Chhurpi"[Title/Abstract] OR "Pe poke"[Title/Abstract] OR "Peruyaan"[Title/Abstract] OR "Peron Naming"[Title/Abstract] OR "Pheha Shoyu"[Title/Abstract] OR "Sieng"[Title/Abstract] OR "Tempeh"[Title/Abstract] OR "Burong mustala"[Title/Abstract] OR "Dha muoi"[Title/Abstract] OR "Fu-tsai"[Title/Abstract] OR "Hom-dong"[Title/Abstract] OR "Hiring"[Title/Abstract] OR "Jiang-gua"[Title/Abstract] OR "Jiang-sun"[Title/Abstract] OR "Naw-mai-dong"[Title/Abstract] OR "Pak-gard-dong"[Title/Abstract] OR "Pak-sian-dong"[Title/Abstract] OR "Suan-cai"[Title/Abstract] OR "Suan-tsai"[Title/Abstract] OR "Takuan-zuke"[Title/Abstract] OR "Miang"[Title/Abstract] OR "Aarak"[Title/Abstract] OR "Angoori"[Title/Abstract] OR "Apong"[Title/Abstract] OR "Basi"[Title/Abstract] OR "Brem"[Title/Abstract] OR "Bhaati jaanr"[Title/Abstract] OR "Baijiu"[Title/Abstract] OR "Chulli"[Title/Abstract] OR "Chyang"[Title/Abstract] OR "Daru"[Title/Abstract] OR "Darassun"[Title/Abstract] OR "Duizou"[Title/Abstract] OR "Ennog"[Title/Abstract] OR "Feni"[Title/Abstract] OR "Handia"[Title/Abstract] OR "Kanji"[Title/Abstract] OR "Khao maak"[Title/Abstract] OR "Kodo ko jaanr"[Title/Abstract] OR "Lugri"[Title/Abstract] OR "Madhu"[Title/Abstract] OR "Mingri"[Title/Abstract] OR "Makgeolli"[Title/Abstract] OR "Poko"[Title/Abstract] OR "Pona"[Title/Abstract] OR "Ruou de"[Title/Abstract] OR "Ruou nep"[Title/Abstract] OR "Raksi"[Title/Abstract] OR "Saké"[Title/Abstract] OR "Sake"[Title/Abstract] OR "Sato"[Title/Abstract] OR "Soju"[Title/Abstract] OR "Shochu"[Title/Abstract] OR "Toddy Takju"[Title/Abstract] OR "Tapuy"[Title/Abstract] OR "Tapai pulut"[Title/Abstract] OR "Tapai ubi"[Title/Abstract] OR "Tapai ubi"[Title/Abstract] OR "Tapé-kekan"[Title/Abstract] OR "Tien-chiu-niang"[Title/Abstract] OR "Yakju"[Title/Abstract] OR "Zutho"[Title/Abstract] OR "Sapal"[Title/Abstract] OR "Chorizo"[Title/Abstract] OR "Morcilla"[Title/Abstract] OR "Peperoni"[Title/Abstract] OR "Saucisson"[Title/Abstract] OR "Rakfisk"[Title/Abstract] OR "Hákarl"[Title/Abstract] OR "Bagni"[Title/Abstract] OR "Kyass"[Title/Abstract] OR "Kvass"[Title/Abstract] OR "Coalho"[Title/Abstract] OR "Corrientes"[Title/Abstract] OR "Minas"[Title/Abstract] OR "Pategrás"[Title/Abstract] OR "Reggianito Argentino Serrano"[Title/Abstract] OR "Calugi"[Title/Abstract] OR "Cauim"[Title/Abstract] OR "Caxiri"[Title/Abstract] OR "Puba"[Title/Abstract] OR "Carimã"[Title/Abstract] OR "Tarubá"[Title/Abstract] OR "Tucupi"[Title/Abstract] OR "Yakupa"[Title/Abstract] OR "Y Parakari"[Title/Abstract] OR "Parakari"[Title/Abstract] OR "Cachaça Chicha"[Title/Abstract] OR "Champu"[Title/Abstract] OR "Masato Pulque"[Title/Abstract] OR "Masato"[Title/Abstract] |
| #2 | "Diet"[Mesh] OR "Life Style"[Mesh] OR "Eating"[Mesh] OR "Feeding Behavior"[Mesh] OR ((food[tiab] OR macronutrient*[tiab] OR eating[tiab]) AND (intake*[tiab] OR habit*[tiab] OR behavior*[tiab] OR pattern*[tiab])) OR diet*[tiab] OR intake[tiab] OR ingestion[tiab] OR suppl*[tiab] OR consumption[tiab] OR meal*[tiab] OR nutrient*[tiab] OR nutrit*[tiab] |
| #3 | #1 AND #2 |
| #4 | "Diet Surveys"[Mesh] OR "Cohort Studies"[Mesh] OR cohort*[Tiab] OR prospective[Tiab] OR longitudinal[Tiab] |
| #5 | Randomized Controlled Trial[Publication Type] OR Controlled Clinical Trial[Publication Type] OR Pragmatic Clinical Trial[Publication Type] OR Clinical Study[Publication Type] OR Adaptive Clinical Trial[Publication Type] OR Equivalence Trial[Publication Type] OR Clinical Trial[Publication Type] OR Clinical Trial, Phase I[Publication Type] OR Clinical Trial, Phase II[Publication Type] OR Clinical Trial, Phase III[Publication Type] OR Clinical Trial, Phase IV[Publication Type] OR Clinical Trial Protocol[Publication Type] OR multicenter study[Publication Type] OR "Clinical Studies as Topic"[Mesh] OR "Clinical Trials as Topic"[Mesh] OR "Clinical Trial Protocols as Topic"[Mesh] OR "Multicenter Studies as Topic"[Mesh] OR "Random Allocation"[Mesh] OR "Double-Blind Method"[Mesh] OR "Single-Blind Method"[Mesh] OR "Placebos"[Mesh:NoExp] OR "Control Groups"[Mesh] OR "Cross-Over Studies"[Mesh] OR random*[Title/Abstract] OR sham[Title/Abstract] OR placebo*[Title/Abstract] OR ((singl*[Title/Abstract] OR doubl*[Title/Abstract]) AND (blind*[Title/Abstract] OR dumm*[Title/Abstract] OR mask*[Title/Abstract])) OR ((tripl*[Title/Abstract] OR trebl*[Title/Abstract]) AND (blind*[Title/Abstract] OR dumm*[Title/Abstract] OR mask*[Title/Abstract])) OR "control study"[tiab:~3] OR "control studies"[tiab:~3] OR "control group"[tiab:~3] OR "control groups"[tiab:~3] OR "healthy volunteers"[tiab:~3] OR "control trial"[tiab:~3] OR "control trials"[tiab:~3] OR "controlled study"[tiab:~3] OR "controlled trial"[tiab:~3] OR "controlled studies"[tiab:~3] OR "controlled trials"[tiab:~3] OR "clinical study"[tiab:~3] OR "clinical studies"[tiab:~3] OR "clinical trial"[tiab:~3] OR "clinical trials"[tiab:~3] OR Nonrandom*[Title/Abstract] OR non random*[Title/Abstract] OR non-random*[Title/Abstract] OR quasi-random*[Title/Abstract] OR quasirandom*[Title/Abstract] OR "phase study"[tiab:~3] OR "phase studies"[tiab:~3] OR "phase trial"[tiab:~3] OR "phase trials"[tiab:~3] OR "crossover study"[tiab:~3] OR "crossover studies"[tiab:~3] OR "crossover trial"[tiab:~3] OR "crossover trials"[tiab:~3] OR "cross-over study"[tiab:~3] OR "cross-over studies"[tiab:~3] OR "cross-over trial"[tiab:~3] OR "cross-over trials"[tiab:~3] OR ((multicent*[tiab] OR multi-cent*[tiab] OR open label[tiab] OR open-label[tiab] OR equivalence[tiab] OR superiority[tiab] OR non-inferiority[tiab] OR noninferiority[tiab] OR quasiexperimental[tiab] OR quasi-experimental[tiab]) AND (study[tiab] OR studies[tiab] OR trial*[tiab])) OR allocated[tiab] OR pragmatic study[tiab] OR pragmatic studies[tiab] OR pragmatic trial*[tiab] OR practical trial*[tiab] |
| #6 | "Epidemiologic Methods"[Mesh:NoExp] OR "Epidemiologic Studies"[Mesh] OR "Observational Studies as Topic"[Mesh] OR "Clinical Studies as Topic"[Mesh] OR "Single-Case Studies as Topic"[Mesh] OR "Organizational Case Studies"[Mesh] OR observational study[Publication Type] OR validation study[Publication Type] OR clinical study[Publication Type] OR case reports[Publication Type] OR "observational study"[tiab:~3] OR "observational studies"[tiab:~3] OR "observational design"[tiab:~3] OR "observational analysis"[tiab:~3] OR "observational analyses"[tiab:~3] OR ((cohort*[tiab] OR prospective[tiab] OR follow-up[tiab] OR longitudinal[tiab] OR long-term[tiab] OR retrospective[tiab]) AND (study[tiab] OR studies[tiab] OR design[tiab] OR analysis[tiab] OR analyses[tiab] OR data[tiab] OR review[tiab])) OR case control*[tiab] OR case comparison*[tiab] OR case-referent[tiab] OR "population study"[tiab:~3] OR "population studies"[tiab:~3] OR "population analysis"[tiab:~3] OR "population analyses"[tiab:~3] OR "descriptive study"[tiab:~3] OR "descriptive studies"[tiab:~3] OR "descriptive design"[tiab:~3] OR "descriptive analysis"[tiab:~3] OR "descriptive analyses"[tiab:~3] OR "multidimensional study"[tiab:~3] OR "multidimensional studies"[tiab:~3] OR "multidimensional design"[tiab:~3] OR "multidimensional analysis"[tiab:~3] OR "multidimensional analyses"[tiab:~3] OR "cross-sectional study"[tiab:~3] OR "cross-sectional studies"[tiab:~3] OR "cross-sectional design"[tiab:~3] OR "cross-sectional analysis"[tiab:~3] OR "cross-sectional analyses"[tiab:~3] OR "cross-sectional research"[tiab:~3] OR "cross-sectional survey"[tiab:~3] OR "cross-sectional findings"[tiab:~3] OR natural experiment*[tiab] OR quasi experiment*[tiab] OR "nonexperimental study"[tiab:~3] OR "nonexperimental studies"[tiab:~3] OR "nonexperimental design"[tiab:~3] OR "nonexperimental analysis"[tiab:~3] OR "nonexperimental analyses"[tiab:~3] OR "prevalence study"[tiab:~3] OR "prevalence studies"[tiab:~3] OR "prevalence analysis"[tiab:~3] OR "prevalence analyses"[tiab:~3] OR case series[tiab] OR "case report"[tiab:~3] OR "case reports"[tiab:~3] OR "case study"[tiab:~3] OR "case studies"[tiab:~3] OR "case histories"[tiab:~3] |
| #7 | "systematic review" |
| #8 | #4 OR #5 OR #6 OR #7 |
| #9 | #3AND #8 |
| #10 | #9 NOT (("Animals"[Mesh] OR "Animal Experimentation"[Mesh] OR "Models, Animal"[Mesh] OR "Vertebrates"[Mesh]) NOT ("Humans"[Mesh] OR "Human Experimentation"[Mesh])) |
| #11 | #10 NOT ("Breast Feeding"[Majr] OR "Milk, Human"[Majr]) |
| #12 | #11 AND (English[Filter]) |
| #13 | #12 AND (("1970/01/01"[Date - Publication] : "2023/08/31"[Date - Publication])) |
| **Scopus** | |
| #1 | TITLE-ABS-KEY ( "Atole" OR "Banku" OR "Bhattejaanr" OR "Burukutu" OR "Jinhua" OR "Skyr" OR "Kaymak" OR "Kumis" OR "Koumiss" OR "Ambra di Talamello" OR "Shanklish" OR "Kajmak" OR "Ayran" OR "Doogh" OR "Buttermilk" OR "Amasi" OR "Filmjölk" OR "Matzoon" OR "Matsoni" OR "Mursik" OR "Viili" OR "Malai" OR "Leben" OR "Yakult" OR "Lassi" OR "Calpis" OR "Chal" OR "Airag" OR "Chhurpi" OR "Dahi" OR "Dadih" OR "Laban rayeb" OR "Lben" OR "Misti dahi" OR "Mishti doi" OR "Lal dahi" OR "Payodhi" OR "Nunu" OR "Philu" OR "Shrikhand" OR "Somar" OR "Sua chua" OR "Tarag" OR "Borhani" OR "Bagoong" OR "Dayok" OR "Ganjang" OR "Garum" OR "Burong Hipon Tagbilao" OR "Jeotgal" OR "Alheira" OR "Androlla" OR "Arjia" OR "Chartayshya" OR "Kargyong" OR "Nham" OR "Musom" OR "Pastirma" OR "Sai Krok Prew" OR "Sai krok Isan" OR "Salchichon" OR "Salsiccia" OR "Soppressata" OR "Sucuk" OR "Suka ko masu" OR "Tocino" OR "Belacan" OR "Blacan" OR "Bakasang" OR "Burong Bangus" OR "Burong Isda" OR "Budu" OR "Gnuchi" OR "Gulbi" OR "Hentak" OR "Hoi-malaeng pu-dong" OR "Jeotgal" OR "Saeoo Jeot" OR "Karati" OR "Bordia" OR "Lashim" OR "Kusaya" OR "Myeolchi jeot" OR "Narezushi" OR "Nam pla" OR "Ngari" OR "Nuoc mam" OR "Patis" OR "Pla paeng Daeng" OR "Plaa Som" OR "Pla khao sug" OR "Shidal" OR "Shottsuru" OR "Sik hae" OR "Suka ko maacha" OR "Sukuti" OR "Surströmming" OR "Tungtap" OR "Caper" OR "Yongfeng chili sauce" OR "Poi" OR "Pao cai" OR "Burong Mustasa" OR "Dhamuoi" OR "Ekung" OR "Eup" OR "Goyang" OR "Gundruk" OR "Khalpi" OR "Mesu" OR "Oiji" OR "Naw Mai Dong" OR "Pak Gad Dong" OR "Sayur asin" OR "Soibum" OR "Soidon" OR "Sinki" OR "Sunki" OR "Takuan" OR "Tuaithur" OR "Bikalga" OR "Masaura" OR "Masyaura" OR "Chikwangue" OR "Fufu" OR "Gari" OR "Lafun" OR "Konkonte" OR "Cheonggukjang" OR "Doenjang" OR "Doubanjiang" OR "Douchi" OR "Pon ye gyi" OR "Ssamjang" OR "Tương" OR "Tungrymbai" OR "Huáng Jiàng" OR "Gochujang" OR "Iru" OR "Sumbala" OR "Ogiri" OR "Ogili" OR "Oncom" OR "Dhokla" OR "Lufu" OR "Jang" OR "Bekang" OR "Chungkokjang" OR "Jeonkukjang" OR "Dawadawa" OR "Furu" OR "Sufu" OR "Hawaijar" OR "Kanjang" OR "Kawal" OR "Kecap" OR "Ketjap" OR "Kinema" OR "Meitauza" OR "Meju" OR "Oncom" OR "Okpehe" OR "Soumbala" OR "Shoyu" OR "Tauco" OR "Thua nao" OR "Ugba" OR "Wari" OR "Yandou" OR "Trachana" OR "Tarhana" OR "Eftazimo" OR "Tianmianjiang" OR "Murri" OR "Appam" OR "Chakuli pitha" OR "Dosa" OR "Enduri Pitha" OR "Idli" OR "Injera" OR "Enjera" OR "Injera Gowé" OR "Kenkey" OR "Khanom chin" OR "Kuzhi paniyaram" OR "Mixian" OR "Ogi" OR "Akamu" OR "Idly" OR "Amazake" OR "Chicha" OR "Boza" OR "Bushera" OR "Busa" OR "Ben saalga" OR "Hussuwa" OR "Jalebi" OR "Khamak" OR "Kao mak" OR "Kunu zaki" OR "Kisra" OR "Koko" OR "Mbege" OR "Pito" OR "Poto poto" OR "Puto" OR "Selroti" OR "Sel Roti" OR "Ketan" OR "Togwa" OR "Uji" OR "Tapai" OR "Tapay" OR "Bánh cuốn" OR "Pozol" OR "Rabadi" OR "Kimchi" OR "Tempe" OR "Miso" OR "Natto" OR "Amabere" OR "Amaruranu" OR "Ergo" OR "Fènè" OR "Fene" OR "Gariss" OR "Kule Naoto" OR "Mabisi" OR "Mafi" OR "Masai" OR "Mutandabota" OR "Omashikwa" OR "Pendidam" OR "Nyarmie" OR "Sethemi" OR "Suusac" OR "Zabady" OR "Ben-saalga" OR "Gowé" OR "Kunu-zaki" OR "Mawè" OR "Basterma" OR "Basturma Gueddid" OR "Khlii" OR "Khlia" OR "Msrana" OR "Merguez" OR "Naqaneq" OR "Pastrami" OR "Sujuk" OR "Soudjouk" OR "Feseekh" OR "Momone" OR "Kinda" OR "Cingwada" OR "Bantu beer" OR "Bouza" OR "Bussa" OR "Kachasu" OR "Mangisi" OR "Merrisa" OR "Sifanu" OR "Tchoukoutou" OR "Chhu" OR "Churkam" OR "Kalari" OR "Mar" OR "Mohi" OR "Phiu" OR "Ang-kak" OR "Jalabi" OR "Lao-chao" OR "Nan" OR "Tape Ketan" OR "Khyopeh" OR "Nem-chua" OR "Sai-krok-prieo" OR "Sa-um" OR "Satchu" OR "Tocin" OR "Balao-Balao" OR "Hoi-Malaeng" OR "Ika-Shiokara" OR "Jeotkal" OR "Myulchijeot" OR "Nampla-Dee" OR "Nampla-Sod" OR "Pla-Paeng-Daeng" OR "Pla-Som" OR "Pla-Khao-Sug" OR "Pu-Dong" OR "Sheedal" OR "Sidra" OR "Sikhae" OR "Bhallae" OR "Maseura" OR "Oncom Hitam" OR "Oncom Merah" OR "Papad" OR "Axone" OR "Aakhoni" OR "Grep Chhurpi" OR "Pe poke" OR "Peruyaan" OR "Peron Naming" OR "Pheha Shoyu" OR "Sieng" OR "Tempeh" OR "Burong mustala" OR "Dha muoi" OR "Fu-tsai" OR "Hom-dong" OR "Hiring" OR "Jiang-gua" OR "Jiang-sun" OR "Naw-mai-dong" OR "Pak-gard-dong" OR "Pak-sian-dong" OR "Suan-cai" OR "Suan-tsai" OR "Takuan-zuke" OR "Miang" OR "Aarak" OR "Angoori" OR "Apong" OR "Basi" OR "Brem" OR "Bhaati jaanr" OR "Baijiu" OR "Chulli" OR "Chyang" OR "Daru" OR "Darassun" OR "Duizou" OR "Ennog" OR "Feni" OR "Handia" OR "Kanji" OR "Khao maak" OR "Kodo ko jaanr" OR "Lugri" OR "Madhu" OR "Mingri" OR "Makgeolli" OR "Poko" OR "Pona" OR "Ruou de" OR "Ruou nep" OR "Raksi" OR "Saké" OR "Sake" OR "Sato" OR "Soju" OR "Shochu" OR "Toddy Takju" OR "Tapuy" OR "Tapai pulut" OR "Tapai ubi" OR "Tapai ubi" OR "Tapé-kekan" OR "Tien-chiu-niang" OR "Yakju" OR "Zutho" OR "Sapal" OR "Chorizo" OR "Morcilla" OR "Peperoni" OR "Saucisson" OR "Rakfisk" OR "Hákarl" OR "Bagni" OR "Kyass" OR "Kvass" OR "Coalho" OR "Corrientes" OR "Minas" OR "Pategrás" OR "Reggianito Argentino Serrano" OR "Calugi" OR "Cauim" OR "Caxiri" OR "Puba" OR "Carimã" OR "Tarubá" OR "Tucupi" OR "Yakupa" OR "Y Parakari" OR "Parakari" OR "Cachaça Chicha" OR "Champu" OR "Masato Pulque" OR "Masato" ) |
| #2 | TITLE-ABS-KEY (((food OR *nutrient* OR eating OR nutrit*) W/6 (intake* OR habit* OR behavior* OR pattern* OR consumption OR suppl* OR ingestion)) OR diet* OR meal*) |
| #3 | #1 AND #2  *(add combination of string numbers to the field in advanced search in the field "Combined queries…")* |
| #4 | TITLE-ABS-KEY (random* OR sham OR placebo*) OR TITLE-ABS-KEY ((singl* OR doubl*) W/1 (blind* OR dumm* OR mask*)) OR TITLE-ABS-KEY ((tripl* OR trebl*) W/1 (blind* OR dumm* OR mask*)) OR TITLE-ABS-KEY (control* W/3 (study OR studies OR trial* OR group*)) OR TITLE-ABS-KEY (clinical W/3 (study OR studies OR trial*)) OR TITLE-ABS-KEY (Nonrandom* OR "non random*" OR non-random* OR quasi-random* OR quasirandom*) OR TITLE-ABS-KEY (phase W/3 (study OR studies OR trial*)) OR TITLE-ABS-KEY ((crossover OR cross-over) W/3 (study OR studies OR trial*)) OR TITLE-ABS-KEY ((multicent* OR multi-cent*) W/3 (study OR studies OR trial*)) OR TITLE-ABS (allocated) OR TITLE-ABS-KEY (("open label" OR open-label) W/5 (study OR studies OR trial*)) OR TITLE-ABS-KEY ((equivalence OR superiority OR non-inferiority OR noninferiority) W/3 (study OR studies OR trial*)) OR TITLE-ABS-KEY ("pragmatic study" OR "pragmatic studies") OR TITLE-ABS-KEY ((pragmatic OR practical) W/3 trial*) OR TITLE-ABS-KEY ((quasiexperimental OR quasi-experimental) W/3 (study OR studies OR trial*)) OR TITLE (trial) OR KEY (trial) |
| #5 | TITLE-ABS-KEY (observational W/3 (study OR studies OR design OR analysis OR analyses)) OR TITLE-ABS-KEY (cohort*) OR TITLE-ABS-KEY (prospective W/7 (study OR studies OR design OR analysis OR analyses)) OR TITLE-ABS-KEY (("follow up" OR followup) W/7 (study OR studies OR design OR analysis OR analyses)) OR TITLE-ABS-KEY ((longitudinal OR longterm OR (long W/1 term)) W/7 (study OR studies OR design OR analysis OR analyses OR data)) OR TITLE-ABS-KEY (retrospective W/7 (study OR studies OR design OR analysis OR analyses OR data OR review)) OR TITLE-ABS-KEY ((case W/1 control) OR (case W/1 comparison) OR (case W/1 controlled)) OR TITLE-ABS-KEY (case-referent W/3 (study OR studies OR design OR analysis OR analyses)) OR TITLE-ABS-KEY (population W/3 (study OR studies OR analysis OR analyses)) OR TITLE-ABS-KEY (descriptive W/3 (study OR studies OR design OR analysis OR analyses)) OR TITLE-ABS-KEY ((multidimensional OR (multi W/1 dimensional)) W/3 (study OR studies OR design OR analysis OR analyses)) OR TITLE-ABS-KEY (cross W/1 sectional W/7 (study OR studies OR design OR research OR analysis OR analyses OR survey OR findings)) OR TITLE-ABS-KEY ((natural W/1 experiment) OR (natural W/1 experiments)) OR TITLE-ABS-KEY (quasi W/1 (experiment OR experiments OR experimental)) OR TITLE-ABS-KEY (("non experiment" OR nonexperiment OR "non experimental" OR nonexperimental) W/3 (study OR studies OR design OR analysis OR analyses)) OR TITLE-ABS-KEY (prevalence W/3 (study OR studies OR analysis OR analyses)) OR TITLE-ABS-KEY ("case series") OR TITLE-ABS-KEY (case W/3 (report OR reports OR study OR studies OR histories)) |
| #6 | TITLE-ABS-KEY ("systematic review") |
| #7 | #4 OR #5 OR #6 *(add combination of string numbers to the field in advanced search in the field "Combined queries…")* |
| #8 | #3 AND #7 *(add combination of string numbers to the field in advanced search in the field "Combined queries…")* |
| #9 | (KEY (animal* OR nonhuman)) AND NOT (KEY (human*)) |
| #10 | #8 AND NOT #9  *(add combination of string numbers to the field in advanced search in the field "Combined queries…")* |
|  | *Limit #10 to English using the language filter* |
|  | *Limit #10 to 1970 - 2023 using the Year filter (Range from 1970 to 2023)* |
| **Cochrane Central** | |
| #1 | (“Atole” OR “Banku” OR “Bhattejaanr” OR “Burukutu” OR “Jinhua” OR “Skyr” OR “Kaymak” OR “Kumis” OR “Koumiss” OR “Ambra di Talamello” OR “Shanklish” OR “Kajmak” OR “Ayran” OR “Doogh” OR “Buttermilk” OR “Amasi” OR “Filmjölk” OR “Matzoon” OR “Matsoni” OR “Mursik” OR “Viili” OR “Malai” OR “Leben” OR “Yakult” OR “Lassi” OR “Calpis” OR “Chal” OR “Airag” OR “Chhurpi” OR “Dahi” OR “Dadih” OR “Laban rayeb” OR “Lben” OR “Misti dahi” OR “Mishti doi” OR “Lal dahi” OR “Payodhi” OR “Nunu” OR “Philu” OR “Shrikhand” OR “Somar” OR “Sua chua” OR “Tarag” OR “Borhani” OR “Bagoong” OR “Dayok” OR “Ganjang” OR “Garum” OR “Burong Hipon Tagbilao” OR “Jeotgal” OR “Alheira” OR “Androlla” OR “Arjia” OR “Chartayshya” OR “Kargyong” OR “Nham” OR “Musom” OR “Pastirma” OR “Sai Krok Prew” OR “Sai krok Isan” OR “Salchichon” OR “Salsiccia” OR “Soppressata” OR “Sucuk” OR “Suka ko masu” OR “Tocino” OR “Belacan” OR “Blacan” OR “Bakasang” OR “Burong Bangus” OR “Burong Isda” OR “Budu” OR “Gnuchi” OR “Gulbi” OR “Hentak” OR “Hoi-malaeng pu-dong” OR “Jeotgal” OR “Saeoo Jeot” OR “Karati” OR “Bordia” OR “Lashim” OR “Kusaya” OR “Myeolchi jeot” OR “Narezushi” OR “Nam pla” OR “Ngari” OR “Nuoc mam” OR “Patis” OR “Pla paeng Daeng” OR “Plaa Som” OR “Pla khao sug” OR “Shidal” OR “Shottsuru” OR “Sik hae” OR “Suka ko maacha” OR “Sukuti” OR “Surströmming” OR “Tungtap” OR “Caper” OR “Yongfeng chili sauce” OR “Poi” OR “Pao cai” OR “Burong Mustasa” OR “Dhamuoi” OR “Ekung” OR “Eup” OR “Goyang” OR “Gundruk” OR “Khalpi” OR “Mesu” OR “Oiji” OR “Naw Mai Dong” OR “Pak Gad Dong” OR “Sayur asin” OR “Soibum” OR “Soidon” OR “Sinki” OR “Sunki” OR “Takuan” OR “Tuaithur” OR “Bikalga” OR “Masaura” OR “Masyaura” OR “Chikwangue” OR “Fufu” OR “Gari” OR “Lafun” OR “Konkonte” OR “Cheonggukjang” OR “Doenjang” OR “Doubanjiang” OR “Douchi” OR “Pon ye gyi” OR “Ssamjang” OR “Tương” OR “Tungrymbai” OR “Huáng Jiàng” OR “Gochujang” OR “Iru” OR “Sumbala” OR “Ogiri” OR “Ogili” OR “Oncom” OR “Dhokla” OR “Lufu” OR “Jang” OR “Bekang” OR “Chungkokjang” OR “Jeonkukjang” OR “Dawadawa” OR “Furu” OR “Sufu” OR “Hawaijar” OR “Kanjang” OR “Kawal” OR “Kecap” OR “Ketjap” OR “Kinema” OR “Meitauza” OR “Meju” OR “Oncom” OR “Okpehe” OR “Soumbala” OR “Shoyu” OR “Tauco” OR “Thua nao” OR “Ugba” OR “Wari” OR “Yandou” OR “Trachana” OR “Tarhana” OR “Eftazimo” OR “Tianmianjiang” OR “Murri” OR “Appam” OR “Chakuli pitha” OR “Dosa” OR “Enduri Pitha” OR “Idli” OR “Injera” OR “Enjera” OR “Injera Gowé” OR “Kenkey” OR “Khanom chin” OR “Kuzhi paniyaram” OR “Mixian” OR “Ogi” OR “Akamu” OR “Idly” OR “Amazake” OR “Chicha” OR “Boza” OR “Bushera” OR “Busa” OR “Ben saalga” OR “Hussuwa” OR “Jalebi” OR “Khamak” OR “Kao mak” OR “Kunu zaki” OR “Kisra” OR “Koko” OR “Mbege” OR “Pito” OR “Poto poto” OR “Puto” OR “Selroti” OR “Sel Roti” OR “Ketan” OR “Togwa” OR “Uji” OR “Tapai” OR “Tapay” OR “Bánh cuốn” OR “Pozol” OR “Rabadi” OR “Kimchi” OR “Tempe” OR “Miso” OR “Natto” OR “Amabere” OR “Amaruranu” OR “Ergo” OR “Fènè” OR “Fene” OR “Gariss” OR “Kule Naoto” OR “Mabisi” OR “Mafi” OR “Masai” OR “Mutandabota” OR “Omashikwa” OR “Pendidam” OR “Nyarmie” OR “Sethemi” OR “Suusac” OR “Zabady” OR “Ben-saalga” OR “Gowé” OR “Kunu-zaki” OR “Mawè” OR “Basterma” OR “Basturma Gueddid” OR “Khlii” OR “Khlia” OR “Msrana” OR “Merguez” OR “Naqaneq” OR “Pastrami” OR “Sujuk” OR “Soudjouk” OR “Feseekh” OR “Momone” OR “Kinda” OR “Cingwada” OR “Bantu beer” OR “Bouza” OR “Bussa” OR “Kachasu” OR “Mangisi” OR “Merrisa” OR “Sifanu” OR “Tchoukoutou” OR “Chhu” OR “Churkam” OR “Kalari” OR “Mar” OR “Mohi” OR “Phiu” OR “Ang-kak” OR “Jalabi” OR “Lao-chao” OR “Nan” OR “Tape Ketan” OR “Khyopeh” OR “Nem-chua” OR “Sai-krok-prieo” OR “Sa-um” OR “Satchu” OR “Tocin” OR “Balao-Balao” OR “Hoi-Malaeng” OR “Ika-Shiokara” OR “Jeotkal” OR “Myulchijeot” OR “Nampla-Dee” OR “Nampla-Sod” OR “Pla-Paeng-Daeng” OR “Pla-Som” OR “Pla-Khao-Sug” OR “Pu-Dong” OR “Sheedal” OR “Sidra” OR “Sikhae” OR “Bhallae” OR “Maseura” OR “Oncom Hitam” OR “Oncom Merah” OR “Papad” OR “Axone” OR “Aakhoni” OR “Grep Chhurpi” OR “Pe poke” OR “Peruyaan” OR “Peron Naming” OR “Pheha Shoyu” OR “Sieng” OR “Tempeh” OR “Burong mustala” OR “Dha muoi” OR “Fu-tsai” OR “Hom-dong” OR “Hiring” OR “Jiang-gua” OR “Jiang-sun” OR “Naw-mai-dong” OR “Pak-gard-dong” OR “Pak-sian-dong” OR “Suan-cai” OR “Suan-tsai” OR “Takuan-zuke” OR “Miang” OR “Aarak” OR “Angoori” OR “Apong” OR “Basi” OR “Brem” OR “Bhaati jaanr” OR “Baijiu” OR “Chulli” OR “Chyang” OR “Daru” OR “Darassun” OR “Duizou” OR “Ennog” OR “Feni” OR “Handia” OR “Kanji” OR “Khao maak” OR “Kodo ko jaanr” OR “Lugri” OR “Madhu” OR “Mingri” OR “Makgeolli” OR “Poko” OR “Pona” OR “Ruou de” OR “Ruou nep” OR “Raksi” OR “Saké” OR “Sake” OR “Sato” OR “Soju” OR “Shochu” OR “Toddy Takju” OR “Tapuy” OR “Tapai pulut” OR “Tapai ubi“ OR “Tapai ubi“ OR “Tapé-kekan” OR “Tien-chiu-niang” OR “Yakju” OR “Zutho” OR “Sapal” OR “Chorizo” OR “Morcilla” OR “Peperoni” OR “Saucisson” OR “Rakfisk” OR “Hákarl” OR “Bagni” OR “Kyass” OR “Kvass” OR “Coalho” OR “Corrientes” OR “Minas” OR “Pategrás” OR “Reggianito Argentino Serrano” OR “Calugi” OR “Cauim” OR “Caxiri” OR “Puba” OR “Carimã” OR “Tarubá” OR “Tucupi” OR “Yakupa” OR “Y Parakari” OR “Parakari” OR “Cachaça Chicha” OR “Champu” OR “Masato Pulque” OR “Masato”):ti,ab,kw |
| #2 | (((food OR macronutrient* OR eating) NEAR/6 (intake* OR habit* OR behavior* OR pattern*)) OR diet* OR intake OR ingestion OR suppl* OR consumption OR meal* OR nutrient* OR nutrit*):ti,ab,kw |
| #3 | #1 AND #2 |
|  | *Search with filter "Title Abstract Keyword" |
|  | **Use results from both Cochrane Reviews and Trials for data selection |
